# Novel data-driven subtypes and stages of brain atrophy in the ALS–FTD spectrum

**DOI:** 10.1186/s40035-023-00389-3

**Published:** 2023-12-07

**Authors:** Ting Shen, Jacob W. Vogel, Jeffrey Duda, Jeffrey S. Phillips, Philip A. Cook, James Gee, Lauren Elman, Colin Quinn, Defne A. Amado, Michael Baer, Lauren Massimo, Murray Grossman, David J. Irwin, Corey T. McMillan

**Affiliations:** 1 Penn Frontotemporal Degeneration Center, Department of Neurology, Perelman School of Medicine, University of Pennsylvania, Philadelphia, PA 19104 USA; 2 Department of Clinical Sciences, SciLifeLab, Lund University, 222 42 Lund, Sweden; 3 Penn Image Computing and Science Lab (PICSL), Department of Radiology, Perelman School of Medicine, University of Pennsylvania, Philadelphia, PA 19104 USA; 4 Department of Neurology, Perelman School of Medicine, University of Pennsylvania, Philadelphia, PA 19104 USA; 5 Digital Neuropathology Laboratory, Department of Neurology, Perelman School of Medicine, University of Pennsylvania, Philadelphia, PA 19104 USA

**Keywords:** Amyotrophic lateral sclerosis, Frontotemporal degeneration, Disease heterogeneity, SuStaIn model

## Abstract

**Background:**

TDP-43 proteinopathies represent a spectrum of neurological disorders, anchored clinically on either end by amyotrophic lateral sclerosis (ALS) and frontotemporal degeneration (FTD). The ALS–FTD spectrum exhibits a diverse range of clinical presentations with overlapping phenotypes, highlighting its heterogeneity. This study was aimed to use disease progression modeling to identify novel data-driven spatial and temporal subtypes of brain atrophy and its progression in the ALS–FTD spectrum.

**Methods:**

We used a data-driven procedure to identify 13 anatomic clusters of brain volume for 57 behavioral variant FTD (bvFTD; with either autopsy-confirmed TDP-43 or TDP-43 proteinopathy-associated genetic variants), 103 ALS, and 47 ALS–FTD patients with likely TDP-43. A Subtype and Stage Inference (SuStaIn) model was trained to identify subtypes of individuals along the ALS–FTD spectrum with distinct brain atrophy patterns, and we related subtypes and stages to clinical, genetic, and neuropathological features of disease.

**Results:**

SuStaIn identified three novel subtypes: two disease subtypes with predominant brain atrophy in either prefrontal/somatomotor regions or limbic-related regions, and a normal-appearing group without obvious brain atrophy. The limbic-predominant subtype tended to present with more impaired cognition, higher frequencies of pathogenic variants in *TBK1* and *TARDBP* genes, and a higher proportion of TDP-43 types B, E and C. In contrast, the prefrontal/somatomotor-predominant subtype had higher frequencies of pathogenic variants in *C9orf72* and *GRN* genes and higher proportion of TDP-43 type A. The normal-appearing brain group showed higher frequency of ALS relative to ALS–FTD and bvFTD patients, higher cognitive capacity, higher proportion of lower motor neuron onset, milder motor symptoms, and lower frequencies of genetic pathogenic variants. The overall SuStaIn stages also correlated with evidence for clinical progression including longer disease duration, higher King’s stage, and cognitive decline. Additionally, SuStaIn stages differed across clinical phenotypes, genotypes and types of TDP-43 pathology.

**Conclusions:**

Our findings suggest distinct neurodegenerative subtypes of disease along the ALS–FTD spectrum that can be identified in vivo, each with distinct brain atrophy, clinical, genetic and pathological patterns.

**Supplementary Information:**

The online version contains supplementary material available at 10.1186/s40035-023-00389-3.

## Introduction

TAR DNA-binding protein of ~ 43 kDa (TDP-43) proteinopathies represent a clinicopathologic spectrum anchored clinically on either end by amyotrophic lateral sclerosis (ALS) and frontotemporal degeneration (FTD). ALS is a fatal neurodegenerative disorder characterized by loss of motor neurons in the brain and the spinal cord, leading to muscle weakness, atrophy and ultimately paralysis [1]. Behavioral variant FTD (bvFTD) is the most common subtype of FTD characterized by impairments in behavior, personality, and/or executive function [2, 3]. ALS may additionally exhibit cognitive and behavioral symptoms overlapping with bvFTD [4, 5] and bvFTD can exhibit motor neuron dysfunction consistent with ALS [6]. These two phenotypes can either occur separately or simultaneously, constituting two ends of the spectrum with ALS–FTD lies in between [7]. Pathologically, approximately half of clinical FTD cases are characterized as frontotemporal lobar degeneration with TDP-43 inclusions (FTLD-TDP), and a majority of ALS cases are classified as ALS with TDP-43 inclusions (ALS-TDP) [8]. Genetically, most cases carrying genetic variants, such as chromosome 9 open reading frame 72 (*C9orf72*), progranulin (*GRN*), TANK-binding kinase 1 (*TBK1*), and TAR DNA binding protein (*TARDBP*), exhibit TDP-43 pathology. The ALS–FTD spectrum also presents with genetic heterogeneity with some mutations primarily leading to either ALS or FTD, while others can result in both FTD and ALS [9]. Thus, ALS and FTD have shared and disparate clinical, neuropathological, and genetic features, underscoring multifaceted heterogeneity within the ALS–FTD spectrum. In accordance with preferred nomenclature, we used FTD to refer to the clinical syndrome of “frontotemporal degeneration” and FTLD to refer to the pathological condition “frontotemporal lobar degeneration”. Defining subtypes and elaborating distinct characteristics in the ALS–FTD spectrum capture potential factors driving the heterogeneity of neurodegeneration.

Neurodegenerative diseases display a high degree of inter-individual variation in disease biomarkers, including neuropsychological profiles, neuroimaging features, and molecular biological indicators. Distinct patterns of brain atrophy have been observed along the ALS–FTD spectrum. Regarding clinical phenotypes, bvFTD patients exhibit greater grey matter atrophy in the frontotemporal cortex, insula, thalamus, striatum, hippocampus and amygdala, while ALS patients show more severe atrophy in the motor cortex, pons and brainstem [10, 11]. Different genetic pathogenic variants also result in distinct patterns of brain atrophy in individuals with ALS–FTD spectrum. These patterns vary in severity, progression rate, and affected brain regions. *C9orf72*-related FTD and ALS are associated with higher degree of atrophy extensively in frontal, parietal, occipital, cingulate and insula regions, thalamus and cerebellum compared to sporadic patients [12–19]. *GRN*-FTD patients tend to exhibit greater grey matter volume loss in the frontal cortex [20, 21]. Longitudinal data suggest that patients with pathogenic variants in *GRN* experience faster brain atrophy progression than those with pathogenic variants in *C9orf72*, indicating different rates of pathological progression and fundamental mechanisms associated with different gene variants [12, 22]. Thus, distinct clinical phenotypes and genotypes may account for both spatial and temporal heterogeneity in brain atrophy patterns.

To better understand the spatial and temporal patterns of brain atrophy, an unsupervised machine-learning algorithm called Subtype and Stage Inference (SuStaIn) was developed. This tool can identify distinct subtypes and extract their progression patterns simultaneously [23], unlike previous studies that applied either subtype-only [24–26] or stage-only [27–29] models. A recent study utilized the SuStaIn algorithm to establish a data-driven pathological TDP-43 staging system in ALS, FTLD-TDP, and limbic-predominant age-related TDP-43 encephalopathy neuropathologic change [30]. They identified two subtypes within FTLD-TDP that were cortical-predominant or brainstem-predominant, and two subtypes within ALS that were subcortical-predominant or corticolimbic-predominant. To date, this method has been applied to reconstruct different patterns of sequential disease progression trajectories in TDP-43 proteinopathies [30], FTD [23, 31] and Alzheimer’s disease (AD) [32, 33], providing fundamental insights into the underlying biological processes of these diseases.

In this study, we set out to investigate the complex progression patterns and heterogeneity within earlier stages of the ALS–FTD spectrum, in contrast to late-stage neuropathological studies. To achieve this, we focused on individuals with high likelihood (clinical ALS) or definite (pathology confirmed or with genetic variants) TDP-43 pathology, and we trained a SuStaIn model on baseline cortical and subcortical volume data. Our prior study using the SuStaIn model trained on TDP-43 proteinopathy data had limitations related to the focus on the end-stage of disease and reliance on ordinal pathology ratings [30]. In contrast, this study utilized more quantitative data, the MRI-derived cortical and subcortical volumes that can identify earlier evidence of brain atrophy. We classified individuals into subtypes with different brain atrophy patterns and extracted a full trajectory for each subtype. Furthermore, we examined the differences in clinical phenotypes, genotypes and pathologies across subtypes. We also assessed the effectiveness of the fitted model by analyzing longitudinal brain volumetric data.

## Methods

### Participants

**Fig. 1:**
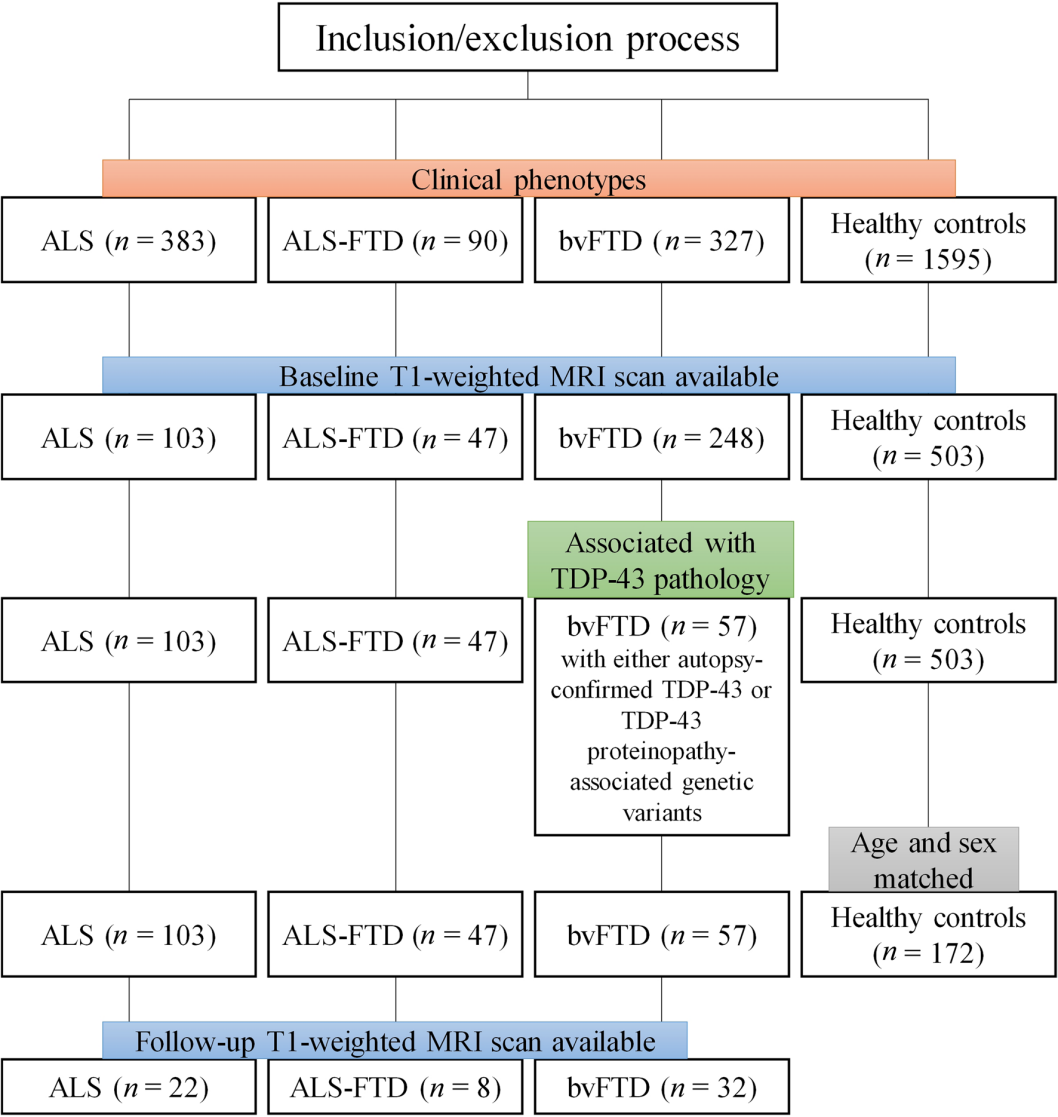
Flow-chart shows the inclusion and exclusion process

Participants were retrospectively selected from the Integrated NeuroDegenerative Disease (INDD) database at the University of Pennsylvania (Fig. 1) [34]. This study included a cohort of individuals who met the published clinical criteria for ALS (*n* = 103), ALS–FTD (*n* = 47), or bvFTD (*n* = 57) [35–37], diagnosed by board-certified neurologists. We also included 172 demographically-comparable (age, sex) healthy controls who self-reported a negative neurological and non-significant psychiatric history with a normal Mini-Mental Status Examination (MMSE) > 27 (out of 30). Individuals with bvFTD had either autopsy-confirmed TDP-43 proteinopathy or genetic evidence of pathogenic variants associated with TDP-43 proteinopathy including *C9orf72*, *GRN*, metalloendopeptidase (*MME*), *TBK1*, and *TARDBP*. Of the 207 individuals with ALS–FTD spectrum disorder, 62 (22 with ALS, 8 with ALS–FTD and 32 with bvFTD) had one follow-up MRI scan, which were used in secondary analyses to evaluate the longitudinal consistency of SuStaIn subtype and stage assignments.

### Neuroimaging data and processing

Structural T1-weighted MRI scans were acquired on a Siemens 3.0 Tesla scanner outfitted as a TIM Trio (*n* = 188) and subsequently as a Prisma Fit (*n* = 81). MRI scans were collected with similar magnetization-prepared rapid gradient-echo (MPRAGE) sequences as follows: (1) 3.0 Tesla Siemens TIM Trio scanner, 8-channel head coil, axial plane with repetition time (TR) ranging from 1620 to 1900 ms, echo time (TE) ranging from 3.09 to 4.38 ms, slice thickness = 1.0 or 1.5 mm, in-plane resolution = 0.98 mm × 0.98 mm; (2) 3.0 Tesla Siemens TIM Trio scanner, 64-channel head coil, sagittal plane with TR = 2200 ms or 2300 ms, TE ranging from 2.95 to 4.63 ms, slice thickness = 1.0 or 1.2 mm, in-plane resolution = 1.0 mm × 1.0 mm; and (3) 3.0 Tesla Siemens Prisma scanner, 64-channel head coil, sagittal plane with TR = 2400 ms, TE = 1.96 ms, slice thickness = 0.8 mm, in-plane resolution = 0.8 mm × 0.8 mm.

Images were processed using the ANT (Advanced Normalization Tools) software package through standard preprocessing steps, as previously described [38]. Briefly, this procedure included N4 bias field correction, diffeomorphic and symmetric registration to a custom template, brain extraction, and segmentation into six tissue classes (cortical grey matter, subcortical grey matter, deep white matter, CSF, brainstem, and cerebellum) using template-based priors [39]. The custom template was in turn aligned to the MNI152 2009c Asymmetric T1-weighted template. The Schaefer 17-network atlas with 100 cortical parcels [40] and the Melbourne subcortex atlas [41] were warped from the MNI152 space through the custom template to individual space. From each label, volumetric measurement was extracted, normalized by age, sex, and intracranial volume and converted to w-scores relative to healthy controls [42].

**Fig. 2:**
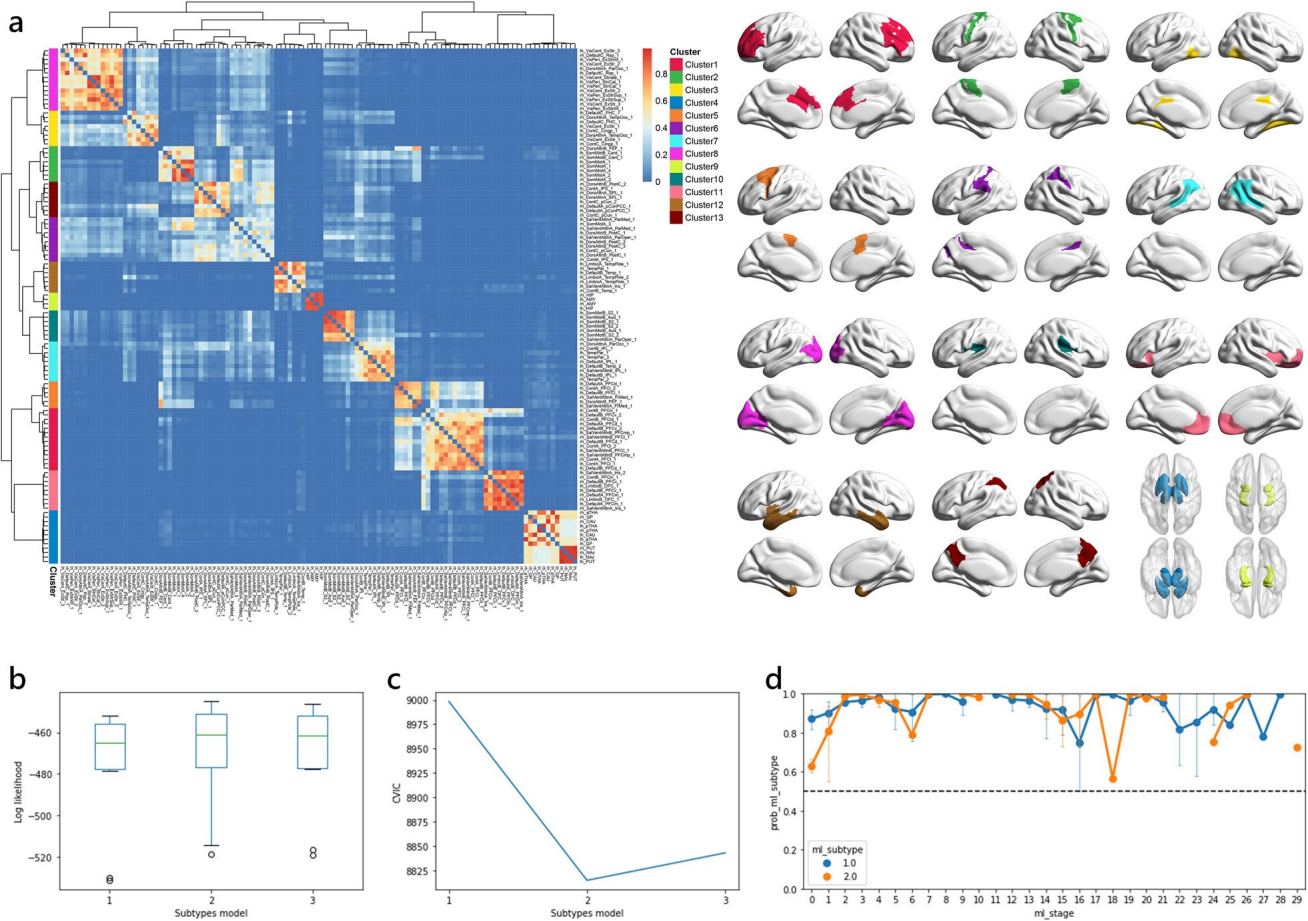
Methodology of selecting optimal number of brain clusters and subtypes. **a** Bootstrap analysis of stable clusters on cortical and subcortical volume. The stability matrix showed that partitions of the brain were classified into stable clusters. **b**, **c** Cross-validation was employed and (**b**) out-of-sample log-likelihood and (**c**) CVIC were both calculated to select the optimal number of subtypes. **d** Subtype probability across SuStaIn stages. *CVIC* cross-validation information criterion

Considering the relatively low dimensionality of input data required for the SuStaIn model, it is important to limit the number of features. We sought data reduction to enhance the power of analysis, improve the model identifiability, and reduce the uncertainty. An unsupervised consensus-clustering algorithm, Bootstrap Analysis of Stable Clusters (BASC), was utilized to identify spatially stable clusters that consistently exhibited similar volumetric measurements of cortical and subcortical structures across subjects [43]. This algorithm performed k-means clustering on 1000 bootstrapped samples to reduce the dimensions of input data. A stability matrix was generated to represent the probabilities of each pair of brain regions falling into the same cluster. Based on the Silhouette index, an optimal number of data-driven clusters were identified. The volumetric measurements of BASC-identified clusters were then extracted and used as input biomarkers to the SuStaIn model (Fig. 2a, Additional file 1: Table S1).

### Clinical data

Clinical and neuropsychological assessments were conducted at the Penn Frontotemporal Degeneration Center and Penn Comprehensive ALS Clinic. Neuropsychological test scores were obtained from the testing visit that was closest to the MRI scan. Demographic information, including age, sex, years of education, disease duration (the time from self- or informant-reported symptom onset to MRI scan), diagnostic delay (the time interval between self- or informant-reported symptom onset and confirmed disease diagnosis) and site of symptom onset, was collected.

#### Motor assessments

The Penn Upper Motor Neuron Score (PUMNS) measures upper motor neuron signs in individuals with ALS/ALS–FTD [44]. The Revised ALS Functional Rating Scale (ALSFRS-R) evaluates the severity of motor symptom functional impairment in ALS/ALS–FTD [45]. Disease progression was measured by the Progression index, which is calculated as (48 – ALSFRS-R score)/duration in months [46]. We also calculated King’s stage, derived from the ALSFRS-R, to assess the spreading of motor symptoms [47].

#### Cognitive assessments

Cognitive and behavioral changes were evaluated using tests, including MMSE, Edinburgh Cognitive Assessment Scale (ECAS) [5, 48], Philadelphia Brief Assessment of Cognition (PBAC) [49], Boston Naming Test (BNT), semantically-guided category naming fluency for the number of animals generated in 60 s (Animal fluency score), letter-guided category naming fluency for the number of ‘F’ words generated in 60 s (Letter fluency score), and digit-span for the longest number of digits repeated in forward and backward sequences (Digit forward span and Digit backward span).

### Genetic screening

Genomic DNA was extracted from peripheral blood or frozen brain tissue collected from participants [50]. DNA was not available from 6 individuals. Genotyping for *C9orf72* hexanucleotide repeat expansions was performed using a modified repeat-primed polymerase-chain reaction, as previously described [51]. Pathogenic variants associated with the ALS–FTD spectrum were screened using either a targeted next-generation sequencing panel (MiND-Seq) [50] or whole-exome/genome (WES/WGS) sequencing. Of the 201 individuals who underwent genetic screening, 64 were found to have pathogenic variants. Specifically, 48 had repeat expansions in *C9orf72* (> 30 repeats), and others had known pathogenic variants including 11 in *GRN*, 1 in *MME*, 2 in *TBK1*, and 2 in *TARDBP*.

### Neuropathological examination

Autopsy was performed on a subset of individuals (*n* = 55) including 21 ALS, 7 ALS–FTD, and 27 bvFTD. Neuropathological diagnosis of FTLD-TDP and ALS-TDP was performed by expert neuropathologists according to previously described protocols [52]. TDP-43 proteinopathies were classified into categories including types A–E [53]. Type A is characterized by abundant neuronal cytoplasmic inclusions (NCIs) and short thick dystrophic neurites (DN) in the superficial cortical layers, with less abundant lentiform neuronal intranuclear inclusions (NIIs). Type B shows moderate numbers of NCI in both superficial and deep cortical layers, with relatively few DN and no NII. Type C has a predominance of long DN in superficial cortical layers, with few NCI and no NII. Type D is typified by lentiform NII and delicate short DN in superficial laminae. Type E exhibits granulofilamentous neuronal cytoplasmic inclusions and grains in both superficial and deep cortical layers. Since type E is relatively rare and shows some biological overlap with type B [53], it has been proposed to combine these two types together. Of the 55 individuals, 16 were classified as type A cases, 18 as type B or E, 3 as type C cases, and the remaining 18 cases (1 bvFTD and 17 ALS) that could not be further subtyped were classified as TDP-43 non-specific type.

### Subtype and stage inference modelling

We utilized the w-scored volumetric measurements of 13 BASC-identified clusters (Fig. 2a, Additional file 1: Table S1) as input biomarkers for training the SuStaIn model (https://github.com/ucl-pond/pySuStaIn). As the volumetric measurements were continuous variables, we employed the piecewise linear SuStaIn model. This algorithm combines clustering and disease progression modelling to identify subtypes with different rates and patterns of disease progression [23]. To evaluate the performance of SuStaIn model, we used 10-fold cross-validation, where the optimal number of subtypes was selected based on the out-of-sample log-likelihood and cross-validation information criterion [23] to better balance the model complexity with accuracy (Fig. 2b, c). Disease progression pattern of each subtype was described by a piecewise linear model, which reconstructed the trajectory of brain atrophy. Each event, alternatively referred to as stage, corresponded to a change in a specific biomarker, quantified by w-scores representing the severity of brain atrophy. We utilized w-score waypoints of 1, 2, and 3, with 3 set as the maximum value that represented the point at which the biomarker reached severe abnormality. To capture the progression pattern where each SuStaIn stage corresponds to a new region reaching a new score, the number of stages was determined by multiplying the number of BASC-identified clusters (13) by the maximum w-score value (3), resulting in a total of 39 stages. The model uncertainty was estimated using 100,000 Markov chain Monte Carlo iterations. For each subject, the SuStaIn model assigned a probability value to each subtype and stage, enabling their assignment to a specific subtype and stage within the disease progression pattern of this subtype.

Longitudinal MRI scans were withheld from the SuStaIn model calculations and then used in a secondary analysis to assess the stability of SuStaIn subtypes and progression of SuStaIn stages over time. At follow-up visits, the volumetric measurements were w-scored as described above using the same healthy control cohort for normalization. Subtype stability was determined as the proportion of individuals who were either assigned to the same subtype or progressed from normal-appearing group to a SuStaIn subtype at follow-up visits. The advancement of SuStaIn stage over time was evaluated in individuals with stable subtypes. The annualized change of SuStaIn stage was calculated by dividing the change in SuStaIn stage from baseline to the follow-up visit by follow-up period.

### Statistical analyses

The statistical analyses and plotting were conducted with R statistical software (version 4.2.0; R Foundation for Statistical Computing, Vienna, Austria) and GraphPad Prism (version 9.0; GraphPad Software, Inc., San Diego, CA). The brain heatmaps were visualized using BrainNet Viewer [54]. The normality of variable distribution was tested using the Shapiro-Wilk normality test. Continuous variables with normal distribution were compared using two-sample *t*-test, while Mann–Whitney test was utilized for comparing variables with non-normal distribution. For comparison of categorical variables, chi-squared test or Fisher exact test was employed. We compared clinical features, frequencies of pathogenic variants, proportions of TDP-43 types, SuStaIn stages and annualized change of SuStaIn stage across subtypes. Additionally, subtype probability at baseline was compared between subtype-stable and unstable individuals. A significance level of *P* < 0.05 was considered significant. Cortical and subcortical volumes were compared between different groups using a generalized linear model, and a false discovery rate (FDR)-corrected *P* < 0.05 was used for multiple testing. Correlation analyses were conducted between the predicted SuStaIn stages and clinical profiles, the baseline and follow-up SuStaIn stages, as well as the change in SuStaIn stage and follow-up period. All correlation analyses were considered significant at a threshold of *P* < 0.05.

## Results

### Participant characteristics

The demographic, clinical, genetic and pathological characteristics of participants are summarized in Table 1. Compared to the ALS individuals, the bvFTD individuals had longer disease duration. The diagnostic delay in individuals with ALS, ALS–FTD, and bvFTD is a multifactorial issue influenced by various elements, and increased in ascending order for these conditions. The ALS individuals were younger and had higher MMSE scores than ALS–FTD and bvFTD individuals. Individuals with bvFTD had higher frequencies of pathogenic variants in *C9orf72* and *GRN* genes than ALS/ALS–FTD, and two individuals with pathogenic *TARDBP* mutations were both bvFTD. The ALS–FTD and bvFTD groups had higher proportions of TDP-43 type A, B, and E cases compared to the ALS group. All three TDP-43 type C cases were bvFTD. Most of the ALS cases in our cohort were classified as TDP-43 non-specific type.

**Table 1:** Comparison of baseline characteristics between clinical phenotypes in all individuals with ALS–FTD spectrum disorder

	ALS *n* = 103	ALS–FTD *n* = 47	bvFTD *n* = 57	Missing data*	*P*_ALSvsALS−FTD_	*P*_ALSvsbvFTD_	*P*_ALS−FTDvsbvFTD_
Age at MRI, years	58.9 (10.3)	62.6 (9.6)	62.9 (7.5)	0.0%	**0.04**	**0.005**	0.85
Sex, male%	58 (56.3%)	28 (59.6%)	36 (63.2%)	0.0%	0.71	0.40	0.71
Education, years	16.7 (12.0)	15.0 (2.8)	16.1 (2.8)	0.0%	0.72	0.09	0.09
Disease duration, months^a^	23.9 (200.1)	28.4 (126.0)	37.3 (201.2)	0.0%	0.20	**0.0004**	0.06
Diagnostic delay, months	17.7 (17.6)	36.8 (34.6)	46.5 (33.2)	0.0%	**< 0.0001**	**< 0.0001**	**0.007**
MMSE	27.7 (2.8)	24.1 (5.7)	24.3 (4.9)	6.3%	**< 0.0001**	**< 0.0001**	0.87
Genetic pathogenic variants^b^	*n* = 99	*n* = 45	*n* = 57	2.9%	**–**	**–**	**–**
*C9orf72*	7 (7.1%)	10 (22.2%)	31 (54.4%)		**0.009**	**< 0.0001**	**0.001**
*GRN*	0 (0.0%)	0 (0.0%)	11 (19.3%)		1.00	**< 0.0001**	**0.002**
*MME*	1 (1.0%)	0 (0.0%)	0 (0.0%)		1.00	1.00	1.00
*TBK1*	0 (0.0%)	1 (2.2%)	1 (1.6%)		0.31	0.37	1.00
*TARDBP*	0 (0.0%)	0 (0.0%)	2 (3.5%)		1.00	0.13	0.50
FTLD/ALS-TDP^c^	*n* = 21	*n* = 7	*n* = 27	73.4%	**0.0003**	**< 0.0001**	0.13
Type A	1 (4.8%)	1 (14.3%)	14 (51.9%)	–	–	–	–
Type B/E	3 (14.3%)	6 (85.7%)	9 (33.3%)	–	–	–	–
Type C	0 (0.0%)	0 (0.0%)	3 (11.1%)	–	–	–	–
Non-specific	17 (81.0%)	0 (0.0%)	1 (3.7%)	–	–	–	–
SuStaIn stage	3.3 (5.2)	12.1 (7.7)	15.6 (7.2)	0.0%	**< 0.0001**	**< 0.0001**	0.02

Bold values indicate statistically significant results

Data are presented as mean (standard deviation) for the continuous variables, and as number (frequency) for the categorical variables

*ALS* amyotrophic lateral sclerosis, *ALS–FTD* amyotrophic lateral sclerosis-frontotemporal degeneration, *bvFTD* behavioral variant frontotemporal degeneration, *MMSE* Mini-Mental Status Examination, *FTLD/ALS-TDP* frontotemporal lobar degeneration or amyotrophic lateral sclerosis with TDP-43 inclusions

*The percentage of individuals with missing data

^a^Data presented as median (range)

^b^Number of individuals for genetic screening

^c^Number of individuals receiving neuropathological examination

### Subtype progression patterns

**Fig. 3:**
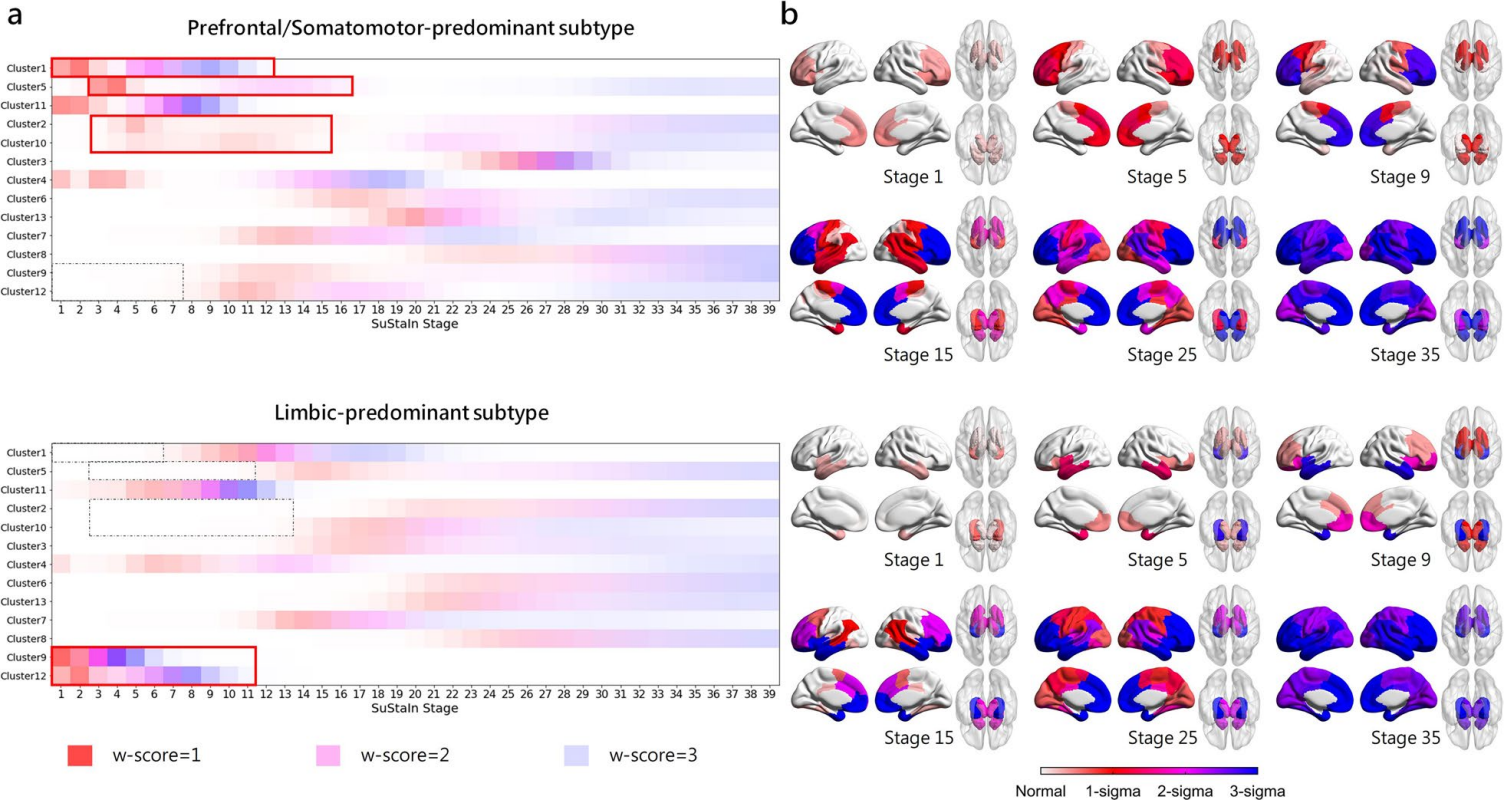
Subtype progression patterns identified by the SuStaIn algorithm. **a** W-scores of subtype progression patterns for each region for each subtype. Color shade represents the probability that w-score in each region is reached at each SuStaIn stage, with red for mild atrophy (w-score = 1), magenta for moderate atrophy (w-score = 2), and blue for severe atrophy (w-score = 3). **b** Spatial distribution and degree of cortical atrophy at each SuStaIn stage. Color shade represents the cumulative sum of probabilities in each brain region

The SuStaIn algorithm was applied to the baseline brain volumetric measurements, resulting in the identification of subtypes that exhibit distinct progression patterns of brain atrophy. Figure 3 illustrates the brain atrophy trajectory for each subtype, with the w-score ranging from 1 to 3, indicating the degree of brain atrophy from mild to moderate to severe. The most noticeable differences between the two subtypes with distinct brain atrophy patterns were observed in the initial sites of brain atrophy during the early SuStaIn stages.

The first identified subtype exhibited brain atrophy that initially appeared in the prefrontal cortex and subsequently in the somatomotor cortex at SuStaIn stage 3, which we subsequently referred to as “Prefrontal/Somatomotor-predominant subtype”. By SuStaIn stage 12–13, parts of the prefrontal cortex reached w-scores exceeding 3. Additionally, the volumetric loss of subcortical regions, including the thalamus, caudate, globus pallidus, putamen, and nucleus accumbens, was evident in early stages but developed more slowly than atrophy in the prefrontal cortex. This volume loss continued to progress and reaches a severe degree after SuStaIn stage 17.

The second identified subtype displayed brain atrophy that was first observed in the temporal pole within the limbic network, hippocampus, and amygdala at SuStaIn stage 1, which we subsequently referred to as “Limbic-predominant subtype”. The brain regions related to the limbic system experienced a more rapid progression of atrophy. Specifically, the hippocampus and amygdala reached w-score 3 by SuStaIn stage 8, while the temporal pole and insula reached w-score 3 by stage 12. The volumetric loss of subcortical regions also began in the early stages of atrophy progression, but it reached w-score 3 later than the Prefrontal/Somatomotor-predominant subtype, indicating a relatively slower rate of progression. It was worth noting that the 11th cluster, which included prefrontal regions, orbitofrontal cortex and insula, experienced significant volumetric loss in the early stages and ultimately reached a severe level of atrophy by SuStaIn stage 11 in both subtypes. In addition to these two subtypes with atrophy, individuals assigned to SuStaIn stage 0 were labeled as “normal-appearing group”, which showed no detectable brain atrophy.

### Subtype assignments

**Fig. 4:**
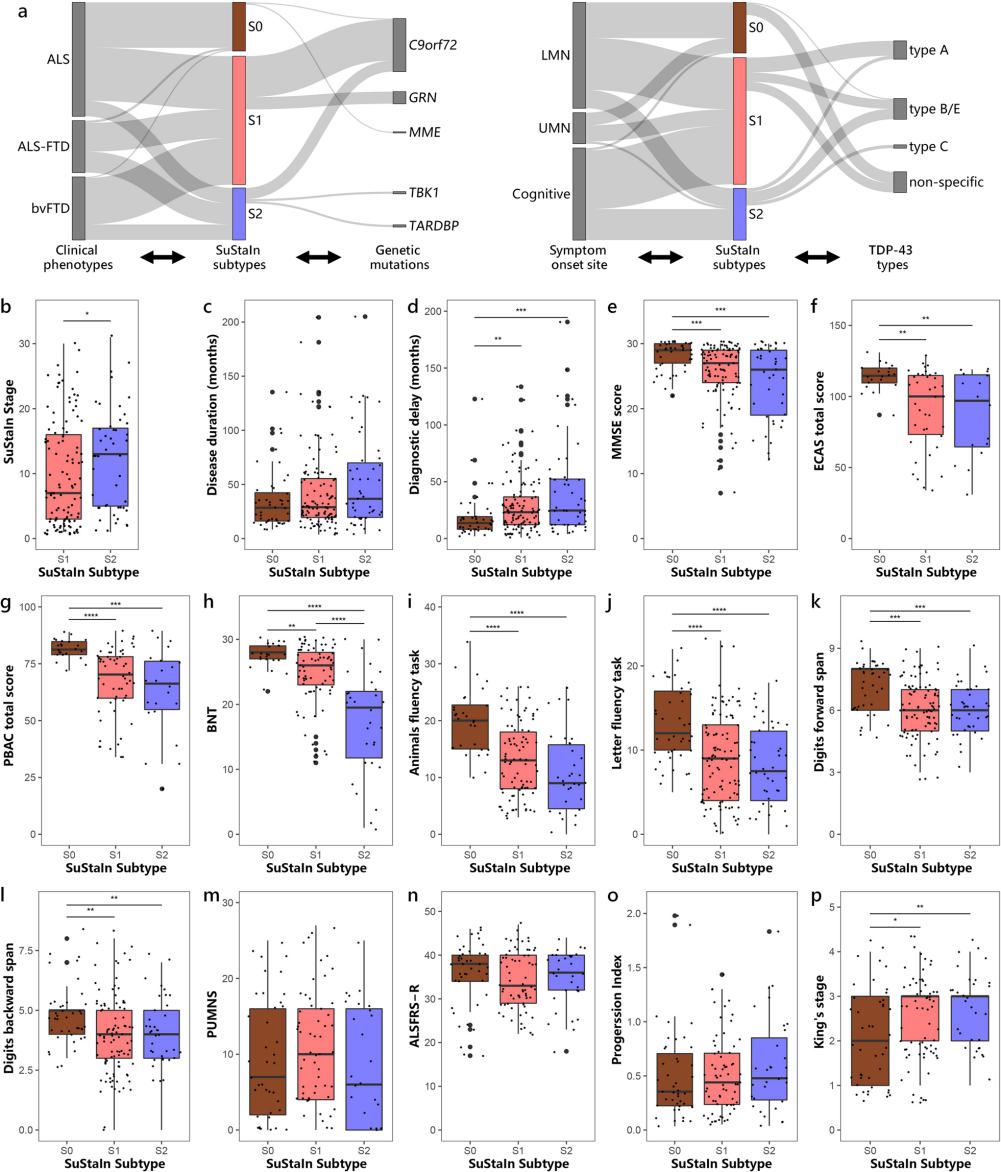
Comparison of clinical, genetic and pathological characteristics across subtypes. **a** Number of clinical phenotypes, cases carrying genetic pathogenic variants, symptom onset sites and TDP-43 types assigned to each subtype. Comparison of **b** SuStaIn stage, **c** disease duration, **d** diagnostic delay, and **e–l** cognitive scores across subtypes in all individuals. Comparison of **m** PUMNS, **n** ALSFRS-R, **o** progression index, and **p** King’s stage across subtypes in individuals with ALS/ALS–FTD. *P < 0.05, **P < 0.01, ****P < 0.0001, ****P < 0.0001. *S0* Normal-appearing group, *S1* Prefrontal/Somatomotor-predominant subtype, *S2* Limbic-predominant subtype, *MMSE* Mini-Mental Status Examination, *ECAS* Edinburgh Cognitive Assessment Scale, *PBAC* Philadelphia Brief Assessment of Cognition, *BNT* Boston naming test, *PUMNS* Penn Upper Motor Neuron Score, *ALSFRS-R* Revised ALS Functional Rating Scale, *LMN* lower motor neuron, *UMN* upper motor neuron

Of individuals with ALS, 48 (46.6%) were categorized as the Prefrontal/Somatomotor-predominant subtype, 14 (13.6%) as the Limbic-predominant subtype, and 41 (39.8%) as the normal-appearing group. The ALS–FTD cohort consisted of 26 (55.3%) individuals classified as the Prefrontal/Somatomotor-predominant subtype, 19 (40.4%) classified as the Limbic-predominant subtype, and 2 (4.3%) categorized as the normal-appearing group. Of individuals with bvFTD, 42 (73.7%) were assigned to the Prefrontal/Somatomotor-predominant subtype, 14 (24.6%) assigned to the Limbic-predominant subtype, and 1 (1.8%) categorized as the normal-appearing group. Thus, individuals with ALS were more likely to be classified into the normal-appearing group, whereas the majority of the ALS–FTD and bvFTD individuals were assigned to atrophy subtypes. The Prefrontal/Somatomotor-predominant subtype was the most common assignment across clinical diagnoses, which had a ~ 1.5-fold higher prevalence compared to the Limbic-predominant subtype. The distribution across subtypes significantly differed among the clinical phenotypes (Fig. 4a, Additional file 1: Table S2).

**Table 2:** Comparison of baseline characteristics between subtypes in all individuals with ALS–FTD spectrum disorder

	S1 *n* = 116	S2 *n* = 47	S0 *n* = 44	Missing data	*P*_S1vsS2_	*P*_S0vS1_	*P*_S0vsS2_
Age at MRI, years	59.9 (9.7)	63.0 (9.5)	60.9 (9.2)	0.0%	0.07	0.59	0.27
Sex, male%	69 (59.5%)	28 (59.6%)	25 (56.8%)	0.0%	0.99	0.76	0.79
Education, years	16.8 (11.3)	15.4 (2.7)	15.1 (2.7)	0.0%	0.80	0.45	0.57
Disease duration, months^a^	29.0 (200.5)	36.7 (200.8)	28.4 (126.9)	0.0%	0.19	0.35	0.08
Diagnostic delay, months	29.1 (24.7)	42.5 (41.8)	19.1 (20.8)	0.0%	0.12	**0.002**	**0.0004**
Clinical phenotypes	*n* = 116	*n* = 47	*n* = 44	0.0%	0.06	**< 0.0001**	**< 0.0001**
ALS	48 (41.4%)	14 (29.8%)	41 (93.1%)	–	–	–	–
ALS–FTD	26 (22.4%)	19 (40.4%)	2 (4.5%)	–	–	–	–
bvFTD	42 (36.2%)	14 (29.8%)	1 (2.3%)	–	–	–	–
Symptom onset site	*n* = 110	*n* = 45	*n* = 44	3.9%	0.17	**< 0.0001**	**< 0.0001**
LMN	45 (40.9%)	16 (35.6%)	31 (70.5%)	–	–	–	–
UMN	14 (12.7%)	2 (4.4%)	11 (25.0%)	–	–	–	–
Cognitive	51 (46.4%)	27 (60.0%)	2 (4.5%)	–	–	–	–
MMSE	25.7 (4.5)	24.3 (5.6)	28.2 (2.2)	6.3%	0.40	**0.0001**	**0.0008**
ECAS total	91.0 (28.0)	87.0 (30.2)	113.9 (9.7)	64.3%	0.75	**0.002**	**0.006**
ALS specific score	66.8 (22.2)	85.5 (8.3)	85.5 (8.3)	–	0.89	**0.001**	**0.009**
ALS non-specific score	24.2 (7.1)	21.8 (7.8)	28.4 (2.5)	–	0.28	**< 0.05**	**0.005**
PBAC total	67.3 (14.9)	62.8 (19.1)	81.5 (4.2)	48.8%	0.31	**< 0.0001**	**0.0002**
Executive scale	8.0 (4.0)	8.8 (3.5)	12.7 (2.5)	–	0.40	**< 0.0001**	**0.0001**
Language scale	16.1 (3.3)	14.2 (4.9)	18.6 (0.6)	–	0.10	**< 0.0001**	**< 0.0001**
Visual scale	15.3 (3.7)	15.9 (3.9)	17.6 (0.7)	–	0.21	**0.0002**	0.05
Memory scale	13.0 (4.7)	10.4 (6.2)	14.6 (3.3)	–	0.13	0.15	**0.02**
Behavioral scale	14.8 (4.0)	13.5 (4.5)	18.0 (0.0)	–	0.15	**< 0.0001**	**< 0.0001**
BNT	24.8 (4.7)	17.3 (8.0)	27.6 (1.9)	37.2%	**< 0.0001**	**0.007**	**< 0.0001**
Animal fluency task	13.1 (6.6)	10.4 (7.1)	19.9 (5.6)	27.5%	0.06	**< 0.0001**	**< 0.0001**
Letter fluency task	8.9 (5.3)	8.0 (5.0)	13.1 (4.5)	6.8%	0.42	**< 0.0001**	**< 0.0001**
Digit forward span	6.1 (1.4)	6.1 (1.2)	7.1 (1.2)	14.0%	0.77	**0.0002**	**0.0003**
Digit backward span	3.9 (1.7)	3.9 (1.6)	4.8 (1.2)	14.5%	0.86	**0.001**	**0.009**
Genetic pathogenic variants^b^	*n* = 112	*n* = 45	*n* = 23	27.1%	**–**	**–**	**–**
*C9orf72*	37 (33.0%)	10 (22.2%)	1 (4.4%)	–	0.18	**0.004**	0.06
*GRN*	11 (9.8%)	0 (0.0%)	0 (0.0%)	–	**0.03**	0.21	1.00
*MME*	0 (0.0%)	0 (0.0%)	1 (4.4%)	–	1.00	0.17	0.34
*TBK1*	0 (0.0%)	2 (4.4%)	0 (0.0%)	–	0.08	1.00	0.55
*TARDBP*	0 (0.0%)	2 (4.4%)	0 (0.0%)	–	0.08	1.00	0.55
FTLD/ALS-TDP^c^	*n* = 29	*n* = 15	*n* = 11	73.4%	**0.002**	**0.0009**	**< 0.0001**
Type A	13 (44.8%)	3 (20.0%)	0 (0.0%)	–	–	–	–
Type B/E	8 (27.6%)	9 (60.0%)	1 (9.1%)	–	–	–	–
Type C	0 (0.0%)	3 (20.0%)	0 (0.0%)	–	–	–	–
Non-specific	8 (27.6%)	0 (0.0%)	10 (90.9%)	–	–	–	–
SuStaIn stage	9.8 (8.1)	12.2 (7.8)	0 (0.0)	0.0%	**< 0.05**	–	–

Bold values indicate statistically significant results

Data are presented as mean (standard deviation) for the continuous variables, and as number (frequency) for the categorical variables. Missing data indicate the percentage of individuals with missing data

*S0* Normal-appearing group, *S1* Prefrontal/Somatomotor-predominant subtype, *S2* Limbic-predominant subtype, *ALS* amyotrophic lateral sclerosis, *ALS–FTD* amyotrophic lateral sclerosis-frontotemporal degeneration, *bvFTD* behavioral variant frontotemporal degeneration, *LMN* lower motor neuron, *UMN* upper motor neuron, *MMSE* Mini-Mental Status Examination, *ECAS* Edinburgh Cognitive Assessment Scale, *PBAC* Philadelphia Brief Assessment of Cognition, *BNT* Boston naming test, *FTLD/ALS-TDP* frontotemporal lobar degeneration or amyotrophic lateral sclerosis with TDP-43 inclusions, *SuStaIn* Subtype and Stage Inference

^a^Data presented as median (range)

^b^Number of individuals for genetic screening

^c^Number of individuals receiving neuropathological examination

### Comparison of cortical and subcortical volumes between subtypes

**Fig. 5:**
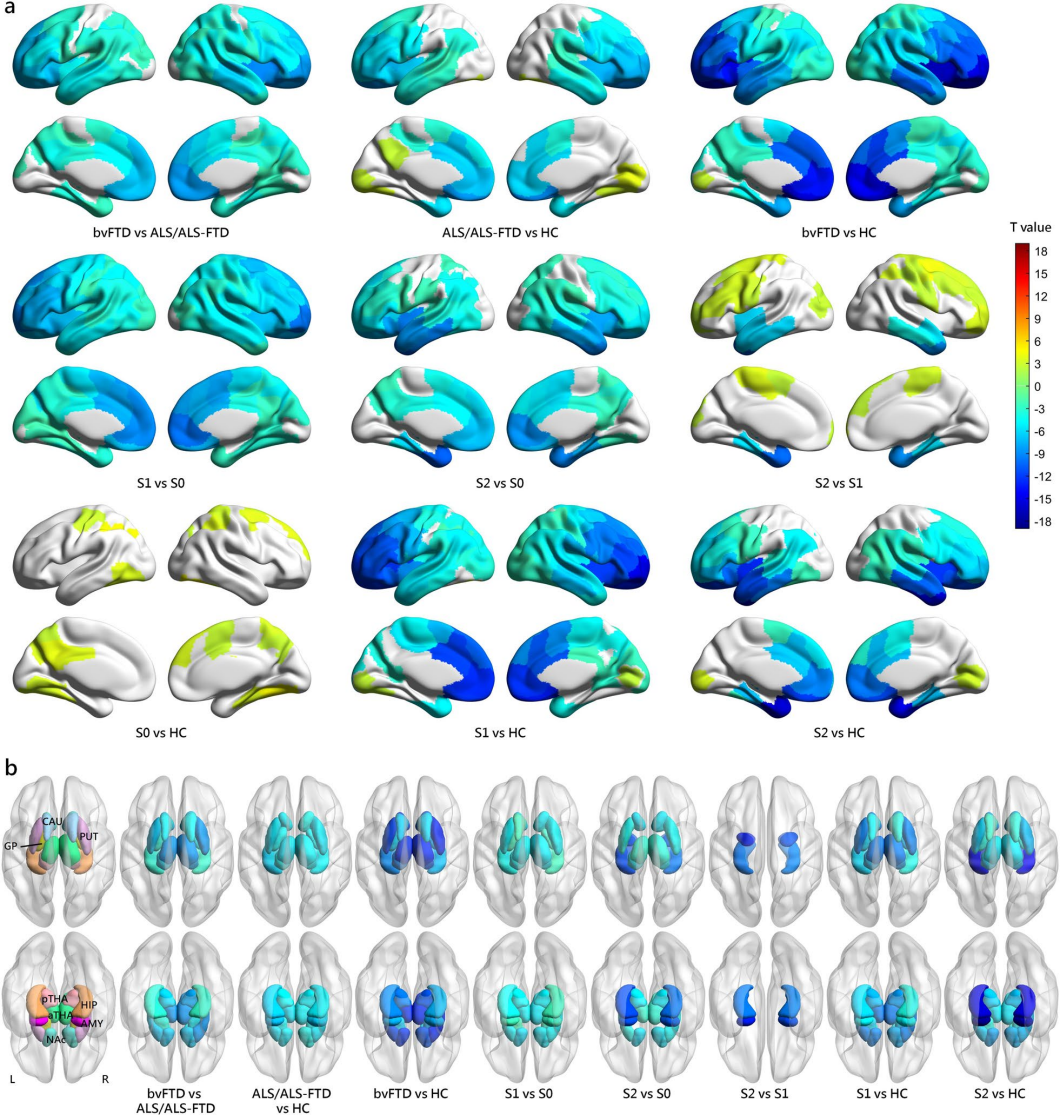
Comparison of volumetric measurements between groups at baseline. **a** Cortical volumetric differences between groups at baseline. **b** Subcortical volumetric differences between groups at baseline. Only results with a threshold at FDR-corrected *P* < 0.05 are shown. Cool colors indicate more cortical atrophy in the former group than the latter one, while warm colors indicate more cortical atrophy in the latter group than the former one. *S0* Normal-appearing group, *S1* Prefrontal/Somatomotor-predominant subtype, *S2* Limbic-predominant subtype

By comparing cortical and subcortical volumes across different groups (Fig. 5), we found that the normal-appearing group did not display any significant brain atrophy at their baseline MRI. As indicated by the name “normal-appearing group”, there was no noticeable reduction of brain volumes compared to healthy controls, which was in line with our expectations.

The two atrophy subtypes displayed extensive decreases of brain volume in comparison to the normal-appearing group. The Prefrontal/Somatomotor-predominant subtype exhibited reduced volume in brain regions within several networks, including somatomotor, limbic, dorsal attention, salience/ventral attention, control, visual, and default mode networks. Additionally, this subtype showed reduced volumes in subcortical regions including thalamus, putamen, globus pallidus, caudate, nucleus accumbens, hippocampus, and amygdala. The Limbic-predominant subtype showed decreased volumes mainly in limbic, dorsal attention, salience/ventral attention, control, and default mode networks, as well as in subcortical regions including hippocampus, amygdala, thalamus, nucleus accumbens and putamen.

The two SuStaIn subtypes exhibited distinct patterns of brain atrophy (Fig. 5). The Limbic-predominant subtype, as indicated by its name, demonstrated lower volumes in the limbic network including temporal pole, insula, parahippocampal cortex, hippocampus, and amygdala relative to the Prefrontal/Somatomotor-predominant subtype. The Prefrontal/Somatomotor-predominant subtype showed lower volumes in prefrontal and somatomotor cortices compared to the Limbic-predominant subtype.

Given the significant difference in SuStaIn stage between subtypes, we conducted additional comparisons of volumetric measurements between subtypes while adjusting for the SuStaIn stage, to avoid attributing regional atrophy differences solely to subtypes with more advanced atrophy due to disease progression (Additional file 1: Fig. S1). Similar findings were observed, more concentrated in regions relevant to the respective subtypes. Specifically, the Prefrontal/Somatomotor-predominant subtype exhibited reduced volume primarily in the thalamus and the prefrontal and somatomotor cortices, while the Limbic-predominant subtype showed decreased volumes mainly in the temporal lobe, the insula, the parahippocampal cortex, the hippocampus, and the amygdala.

### Comparison of clinical, genetic, and neuropathological features between subtypes

Demographic, clinical, genetic and neuropathological characteristics for each subtype are summarized in Fig. 4 and Additional file 1: Table S2. Although the two SuStaIn subtypes displayed different patterns of brain atrophy, there were substantial overlaps in clinical features across subtypes. This suggests that despite differences in neurodegenerative patterns, the clinical manifestations and symptomatology remain largely consistent between the subtypes. The Limbic-predominant subtype exhibited poorer performance in BNT, which assesses language and semantic memory, compared to the Prefrontal/Somatomotor-predominant subtype. In terms of genetic status, the Prefrontal/Somatomotor-predominant subtype had a significantly higher frequency of pathogenic variants in *GRN* compared to the Limbic-predominant subtype. Notably, all 11 cases with *GRN* pathogenic variants were classified into the Prefrontal/Somatomotor-predominant subtype. Although not statistically significant, there was also a trend towards higher frequencies of repeat expansions in *C9orf72* in the Prefrontal/Somatomotor-predominant subtype. Additionally, it is worth highlighting that two individuals with bvFTD who had pathogenic variants in the *TARDBP* gene, as well as one individual with ALS–FTD and one with bvFTD who carried *TBK1* pathogenic variants, were all classified under the Limbic-predominant subtype. Distribution of TDP-43 types varied across SuStaIn subtypes. The Prefrontal/Somatomotor-predominant subtype had a higher proportion of TDP-43 type A. The Limbic-predominant subtype was more prone to TDP-43 type B or E, and all three bvFTD individuals with TDP-43 type C also belonged to this subtype. The TDP-43 non-specific type, predominantly observed in individuals with ALS-TDP, was more prevalent in the Prefrontal/Somatomotor-predominant subtype than in the Limbic-predominant subtype. Compared to the atrophy subtypes, the normal-appearing group had a significantly shorter diagnostic delay, and a higher proportion of individuals with ALS than ALS–FTD and bvFTD. Additionally, they had a lower frequency of cognitive onset in relation to lower and upper motor neuron onset. This group also showed higher cognitive scores, as evidenced by better performance in tests including MMSE, ECAS, PBAC, BNT, Animal and Letter fluency tasks, and Digit forward and backward span. Two cases in the normal-appearing group were found to have pathogenic variants in either *C9orf72* or *MME* gene. Additionally, most individuals in this group who underwent autopsy were classified as having TDP-43 non-specific type pathology.

Certain tests (including PUMNS, ALSFRS-R, Progression index, and King’s stage) were specifically administered for individuals with ALS/ALS–FTD, as these tests were considered more relevant or sensitive in assessing motor impairments. Thus, we focused on ALS/ALS–FTD as a distinct subgroup to compare clinical profiles across subtypes (Fig. 4, Additional file 1: Table S2). Despite a smaller number of ALS–FTD cases in this cohort, the Limbic-predominant subtype still exhibited a higher percentage of individuals with ALS–FTD compared to the Prefrontal/Somatomotor-predominant subtype. Likewise, individuals who experienced cognitive onset were more likely to be classified under the Limbic-predominant subtype, given that this subtype had more individuals with cognitive decline. Regarding the motor symptom scales, the normal-appearing group tended to have lower King’s stages compared to atrophy subtypes. Moreover, by focusing solely on bvFTD (Additional file 1: Table S3), the research sample was relatively homogeneous, allowing for a comprehensive examination of cognitive function across subtypes. The Limbic-predominant subtype had longer disease duration and only showed worse performance in the BNT.

To demonstrate that the differences between two subtypes were related to atrophy patterns rather than one subtype being in a more advanced stage, we further adjusted for SuStaIn stage when comparing the clinical profiles. This adjustment allowed us to account for the potential confounding effect of disease progression. Even after adjusting for SuStaIn stage, the Limbic-predominant subtype still showed poorer performance in the BNT (*t*-statistic = − 5.70, *P* < 0.0001) and on language scale (*t*-statistic = − 2.17, *P* = 0.03) of PBAC compared to the Prefrontal/Somatomotor-predominant subtype. This finding further supported the presence of language impairments in the Limbic-predominant subtype. Furthermore, the Limbic-predominant subtype showed longer diagnostic delay (*t*-statistic = 2.009, *P* = 0.04).

### Relationship between SuStaIn stage and clinical characteristics

**Fig. 6:**
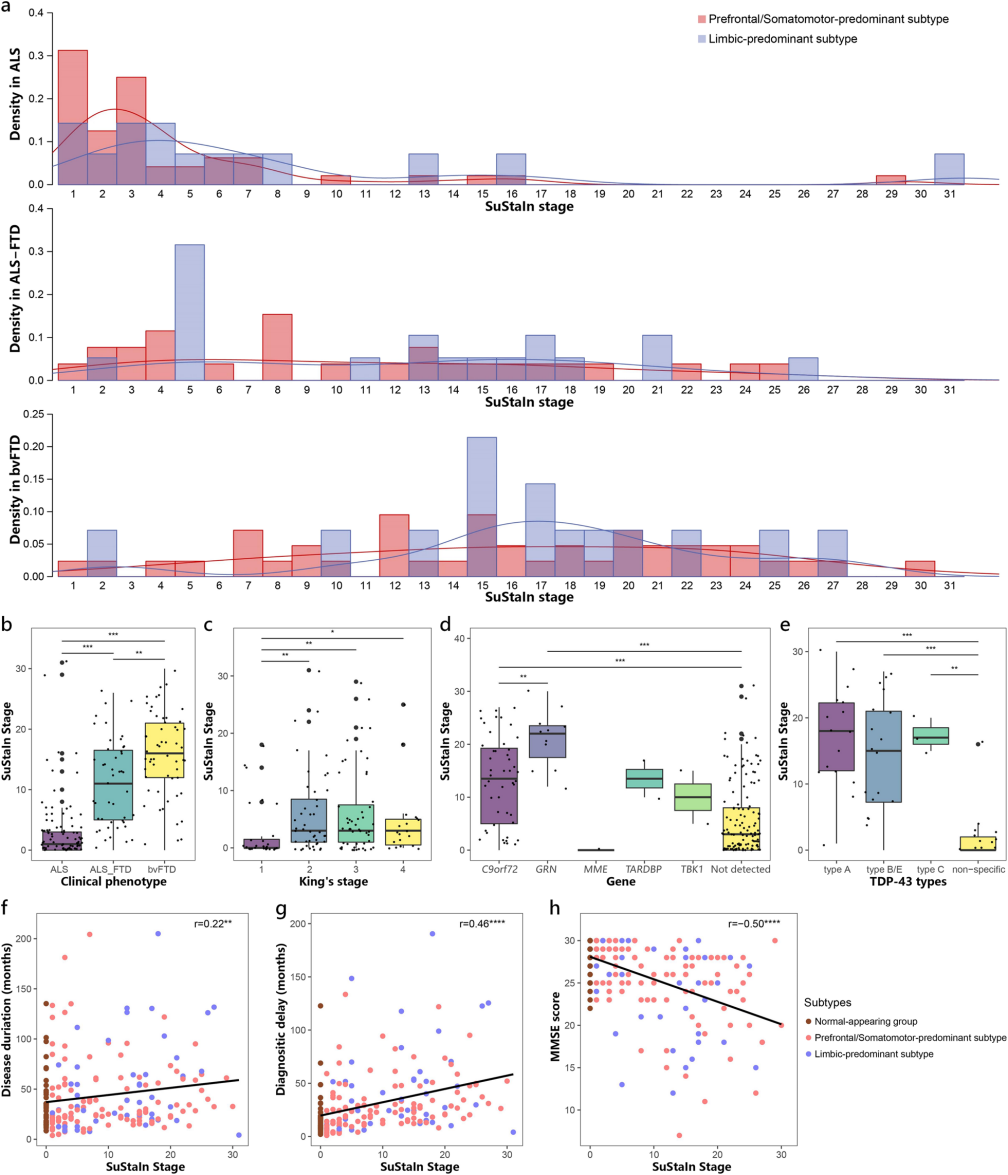
Progression of SuStaIn subtypes. **a** Distribution of individuals assigned to each SuStaIn stage in different clinical phenotypes. **b**–**e** Comparison of SuStaIn stages between different clinical phenotypes (**b**), King’s stages (**c**), genetic pathogenic variants (**d**), and TDP-43 types (**e**). **f**–**h** Increasing SuStaIn stage was correlated with longer disease duration (**f**), longer diagnostic delay (**g**) and worse cognitive function (**h**) across all subtypes. *P < 0.05, **P < 0.01, ****P < 0.0001, ****P < 0.0001

Each individual was assigned to a SuStaIn stage, which reflected progression of brain atrophy. The distribution of individuals assigned to each SuStaIn stage is illustrated in Fig. 6a. ALS individuals were predominantly assigned to earlier SuStaIn stages of brain atrophy, while ALS–FTD and bvFTD individuals were more frequently assigned to later stages (Fig. 6b). Individuals in the Limbic-predominant subtype had higher SuStaIn stages than individuals in the Prefrontal/Somatomotor-predominant subtype (Fig. 4b; Table 2, Additional file 1: Table S2).

We further investigated the relationship between SuStaIn stage and clinical profile, genotype, and neuropathologies in all individuals. The SuStaIn stage was positively correlated with disease duration (*r* = 0.22, *P* = 0.002; Fig. 6f) and diagnostic delay (*r* = 0.46, *P* < 0.0001; Fig. 6g), while negatively correlated with cognitive scales including MMSE (*r* = − 0.50, *P* < 0.0001; Fig. 6h), ECAS scores, PBAC score, BNT, Animal and Letter fluency tasks, and Digit forward and back span tasks (Additional file 1: Fig. S2). In terms of motor symptoms, individuals with ALS/ALS–FTD who had higher King’s stages exhibited higher SuStaIn stages compared to individuals in King’s stage 1 (Fig. 6c). Furthermore, individuals carrying pathogenic variants in *C9orf72* and *GRN* had significantly higher SuStaIn stages, compared to sporadic forms of the disease (Fig. 6d). Individuals with pathogenic variants in *GRN* exhibited higher SuStaIn stages than those who had pathogenic variants in *C9orf72*. Furthermore, autopsy-confirmed TDP-43 typable cases including type A, B, C, and E, also showed significantly higher SuStaIn stages than cases having TDP-43 non-specific type (Fig. 6e).

### Longitudinal stability and reliability of SuStaIn subtypes and stages

#### Subtyping stability

**Fig. 7:**
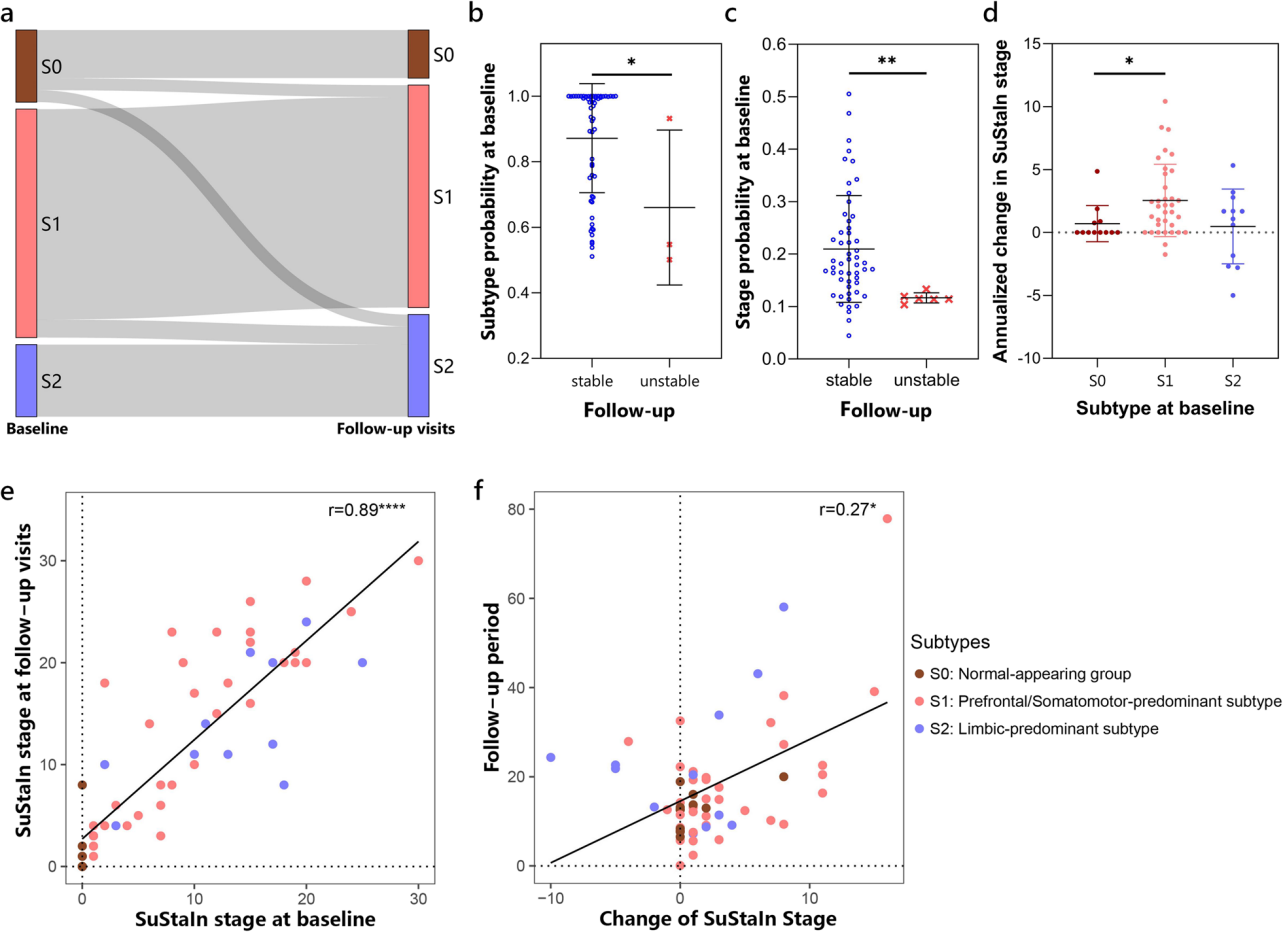
Stability of SuStaIn subtypes. **a** Longitudinal subtype consistency. **b** Subtype probability at baseline in groups of stable or unstable longitudinal subtype assignments. **c** Stage probability at baseline in groups of stable or unstable longitudinal stage assignments. **d** Annualized change in SuStaIn stage of each subtype in individuals with stable subtypes over time. **e** Correlations between SuStaIn stages at baseline and follow-up visits. **f** Correlations between the follow-up period and change of SuStaIn stages. *P < 0.05, **P < 0.01, ****P < 0.0001

The mean follow-up period was 17.5 months, with a standard deviation of 13.1 months. The subtype assignments of follow-up visits are shown in Fig. 7a and Additional file 1: Table S4. Of the 62 follow-up visits, 55 (88.7%) remained consistent with their baseline subtype assignments. Additionally, 2 (3.2%) individuals initially assigned to the normal-appearing group progressed to the Prefrontal/Somatomotor-predominant subtype, while 2 (3.2%) progressed to the Limbic-predominant subtype. These 59 cases (95.2%) were deemed as “subtype stable” individuals. The remaining 3 (4.8%) follow-up visits resulted in inconsistent subtype assignments, and were considered as “subtype unstable”. The probability that each individual belongs to the SuStaIn subtype was estimated. Notably, the probability of subtype assignments at baseline was higher in subtype stable individuals than in unstable individuals (Mann–Whitney U-statistic = 27, *P* = 0.04; Fig. 7b). Individuals assigned to the Prefrontal/Somatomotor-predominant subtype exhibited more atrophy in its key regions, the BASC-identified clusters 1, 2, 5, and 10. The Limbic-predominant subtype showed more atrophy in its key regions, the BASC-identified clusters 9 and 12 (Fig. 3, Additional file 1: Fig. S3). During follow-up visits, brain atrophy showed slight progression. Specifically, the two normal-appearing cases progressing to the Limbic-predominant subtype exhibited significant atrophy progression, particularly in clusters 9 and 12. In contrast, the two normal-appearing cases progressing to the Prefrontal/Somatomotor-predominant subtype showed more widespread atrophy progression, particularly in the prefrontal cortex, with less pronounced progression in the limbic-related regions (Additional file 1: Fig. S3b). Cases displaying abnormal longitudinal changes were typically classified as “subtype unstable” or “stage unstable”.

#### Staging reliability

Among individuals with stable subtype, most of the follow-up visits were assigned to a more advanced SuStaIn stage or remained at the same stage. Of the 59 subtype stable cases, 6 (10.2%) follow-up visits were retrogressed to an earlier stage and regarded as “stage unstable” individuals. The probability of stage assignments at baseline was significantly higher in stage stable individuals compared to unstable individuals (Mann–Whitney U-statistic = 45, *P* = 0.003; Fig. 7c). The annualized change in SuStaIn stage may indicate the rate of disease progression, with the normal-appearing group showing slower progression than the Prefrontal/Somatomotor-predominant subtype (Mann–Whitney U-statistic = 110, *P* = 0.01; Fig. 7d). In stage-stable individuals, annualized change in SuStaIn stage was significantly smaller in the normal-appearing group compared to both atrophy subtypes (Mann–Whitney U-statistic = 86 and *P* = 0.003 for Prefrontal/Somatomotor-predominant subtype, and Mann–Whitney U-statistic = 14 and *P* = 0.005 for Limbic-predominant subtype). Additionally, the SuStaIn stage at baseline was significantly correlated with stages at follow-up visits (*r* = 0.89, *P* < 0.0001; Fig. 7e). Furthermore, we observed a positive correlation between the follow-up period and the change of SuStaIn stage (*r* = 0.27, *P* = 0.04; Fig. 7f).

## Discussion

In this study, we utilized a data-driven SuStaIn model approach to investigate diverse spatial and temporal patterns of brain atrophy in the ALS–FTD spectrum. By analyzing the baseline cross-sectional volumetric imaging data, we identified distinct patterns of regional brain atrophy, which included a Prefrontal/Somatomotor-predominant subtype, a Limbic-predominant subtype and a normal-appearing group. These data-driven subtypes exhibited variations in clinical, genetic and neuropathological characteristics. Moreover, the data-driven SuStaIn stages constructed progression trajectories of each subtype, which aligned with worsening clinical profiles. Together, our findings provide new insights into the heterogeneity in progression patterns of brain atrophy in the ALS–FTD spectrum and highlight the potential utility for patient stratification in precision medicine.

Supporting evidence has demonstrated that the ALS–FTD spectrum displays a high degree of clinical, genetic and neuropathological heterogeneities [10]. Although various biomarkers have been applied to subtype individuals and characterize their brain atrophy patterns within the ALS–FTD spectrum [22, 55, 56], there is still no ideal method to fully disentangle the heterogeneity of brain atrophy. Using the SuStaIn model, we identified data-driven subtypes with distinct progression patterns of brain atrophy. The Prefrontal/Somatomotor-predominant and the Limbic-predominant subtypes exhibited brain atrophy in shared and distinct brain regions. The two subtypes were characterized by their distinctive brain atrophy regions as their names suggest. The Prefrontal/Somatomotor-predominant subtype exhibited atrophy in prefrontal and somatomotor regions, while the Limbic-predominant subtype exhibited atrophy in the limbic-related regions such as temporal regions, hippocampus and amygdala. In addition, both subtypes exhibited volumetric loss in several shared brain regions including prefrontal, paralimbic, and subcortical regions. The prefrontal regions were likely to be the vulnerable regions in the Prefrontal/Somatomotor-predominant subtype, while the orbitofrontal cortex and insula, as two major components of the paralimbic belt, were vulnerable regions in the Limbic-predominant subtype. Our findings were partly consistent with previous studies that have identified subtypes of brain atrophy in subsets of the ALS–FTD spectrum [26, 57]. Tan et al. utilized a subtype-only clustering algorithm and identified subtypes in ALS, one involving motor regions and the other involving orbitofrontal/temporal regions [57]. Bede et al. also identified two distinct subgroups in ALS, one with more motor involvement and one with more frontotemporal pathology [26]. Ranasinghe et al. focused on bvFTD and identified subgroups characterized by predominance in salience network, semantic appraisal network, and subcortical regions [58]. In the present study, we trained the SuStaIn model on a diverse range of clinical phenotypes within the ALS–FTD spectrum. Due to the shared and distinct clinical, neuropathological, and genetic features of ALS and FTD, it is likely that these clinical phenotypes may also possess both overlapping and unique neural foundations. The application of the SuStaIn model allows us to untangle the complexity inherent in these diseases. We were able to identify subtype-specific neural foundations, providing a deeper understanding of disease mechanisms and capturing potential factors driving the inter-individual heterogeneity across the ALS–FTD spectrum. Our approach benefited by considering both spatial and temporal progression of brain atrophy, setting it apart from previous subtype-only and stage-only studies. By incorporating spatial patterns of brain atrophy, we gained a more comprehensive understanding of the different subtypes within the ALS–FTD spectrum. Simultaneously, analysis of temporal progression allowed us to capture the dynamic nature of brain atrophy in the ALS–FTD spectrum, allowing determination of the progressive stage of an individual. As a result, the two subtypes we identified provide a comprehensive summary of the characteristics of previously identified subtypes.

Based on a summary of this study and previous studies [26, 30, 57], we can broadly categorize distinct subtypes with specific disease progression patterns within the TDP-43 proteinopathy spectrum as a frontal/motor-predominant subtype and a frontal/temporal (limbic)-predominant subtype. This approach has also been applied in neurogenerative diseases caused by various proteinopathies. Young et al. utilized the SuStaIn model to individuals with FTD carrying mutations in the *MAPT* gene, and identified two spatiotemporal trajectories of tau spreading in FTLD with tau pathology (FTLD-tau). One of these subtypes, referred to as the temporal subtype, exhibited brain atrophy in the temporal cortex, hippocampus, amygdala and insula. The other, termed as the frontotemporal subtype, displayed atrophy in the lateral temporal lobe, anterior insula, orbitofrontal and ventromedial prefrontal cortex and anterior cingulate [31]. Vogel et al. further applied this model to flortaucipir PET tau images in AD to extract distinct spatiotemporal trajectories of tau spreading [32]. They identified a limbic-predominant subtype, a parietal-dominant and medial temporal lobe-sparing subtype, a posterior occipitotemporal-predominant subtype, and an asymmetric temporoparietal subtype across the AD group. Therefore, across diverse clinical phenotypes and utilizing various neuroimaging techniques, the limbic-predominant subtype is consistently emerging as a distinct subtype, representing one of the discernible patterns of tau pathology spread. Moreover, when incorporating the research on TDP-43 pathology, this limbic-predominant subtype might potentially serve as a shared disease progression trajectory across various neurodegenerative diseases induced by different proteinopathies. In contrast, other subtypes are likely to represent distinct disease progression trajectories unique to various neurodegenerative diseases.

The two brain atrophy subtypes identified in this study showed distinct characteristics. The Limbic-predominant subtype captured a higher proportion of individuals with cognitive (rather than motor) symptom onset, with more pronounced cognitive decline, particularly in the language domain. This subtype resembled a semantic variant primary progressive aphasia pattern. The Prefrontal/Somatomotor-predominant subtype had higher frequencies of pathogenic variants in *C9orf72*. The *C9orf72* pathogenic variant-carriers were demonstrated to exhibit prominent structural and functional disruptions in various brain regions, including prefrontal and motor cortices [59, 60]. Additionally, this subtype also covered all the *GRN* pathogenic variant-carriers. FTD individuals with *GRN* pathogenic variants may exhibit asymmetric cortical atrophy involving frontal, temporal and parietal cortices [12, 61, 62]. Both two bvFTD individuals with the I383V variant in the *TARDBP* gene fell into the Limbic-predominant subtype, consistent with previous observations that the I383V variant is associated with predominant atrophy of temporal lobes and hippocampus [63, 64]. The distribution of TDP-43 types was different between subtypes. The Prefrontal/Somatomotor-predominant subtype had a higher proportion of type A, which has been linked to atrophy in the dorsal frontotemporal, striatal, and thalamic regions [55, 59], all of which were predominant regions of this subtype. The Limbic-predominant subtype presented higher proportions of TDP-43 types B and E. It has been reported that the TDP-43 type B is associated with relatively symmetric atrophy of the medial temporal, medial prefrontal, and orbitofrontal-insular cortices [55], which are regions involved in the Limbic-predominant subtype. The TDP-43 type C is highly associated with neurodegeneration in the anterior temporal lobes including the temporal pole and the amygdalo-hippocampal area [65]. It is notable that all three bvFTD individuals with confirmed TDP-43 type C pathology fell into the Limbic-predominant subtype, which aligns with a staging system of brain atrophy in TDP-43 type C with early involvement of amygdala, medial and lateral temporal cortex, and temporal pole, followed by later involvement of insula [66]. The normal-appearance group displayed better cognitive abilities in various domains including executive functioning, language, visual skill, and memory, as well as milder behavioral symptoms and a tendency towards shorter disease duration. This group mostly consisted of individuals with ALS, who exhibited better cognitive performance and were more likely to be lower-motor-neuron onset. These observations are in line with established knowledge, which suggests that ALS typically exhibits a lesser degree of cortical TDP-43 pathology and greater involvement of lower motor neurons [67]. The spread of TDP-43 pathology in ALS follows a sequential pattern, starting from motor neurons in the spinal cord, brainstem, and agranular motor cortex, then propagating to the frontotemporal and subcortical regions [52].

The SuStaIn model further reconstructed the progression trajectories of brain atrophy of each subtype. The SuStaIn stages represent ordered progression of brain atrophy from normal to a certain degree of abnormality. The Limbic-predominant subtype had higher SuStaIn stages, indicating a more advanced degree of brain atrophy progression than the Prefrontal/Somatomotor-predominant subtype. Individuals with genetic pathogenic variants were assigned to more advanced stages compared to the sporadic individuals. Specifically, the individuals with pathogenic variants in *GRN* exhibited more advanced stages than those with the *C9orf72* repeat expansions. This aligns with previous work demonstrating a faster progression rate of brain atrophy in individuals with pathogenic variants in *GRN* than those in *C9orf72* [22, 68]. Furthermore, individuals with TDP-43 non-specific type exhibited higher SuStaIn stages compared to those with typable TDP-43 pathology. This is because the TDP-43 non-specific type mainly consisted of ALS cases with less cortical pathology, making them unclassifiable into specific TDP-43 types. These individuals exhibited less brain atrophy, indicating an early-stage level of brain atrophy. As individuals entered advanced SuStaIn stages, brain atrophy was increased in degree and spatial extent, accompanied by a subsequent progression of clinical symptoms. SuStaIn stage showed good linear correlations with clinical progression measures including disease duration and cognitive decline. Additionally, regarding motor symptoms, individuals with ALS/ALS–FTD in higher King’s stages exhibited correspondingly higher SuStaIn stages compared to those in King’s stage 1. This finding aligns with a previous study that illustrated the progression of cervical spinal cord atrophy spreading from gray to white matter across King’s stages [69]. Therefore, the spread of TDP-43 pathology could be reflected by these observed relationships, suggesting that as the disease advances in terms of motor and cognitive symptoms, there might be a concurrent progression at the level of neuroanatomic morphological changes. These findings established the SuStaIn stage as a reliable representation of disease progression and could be used to evaluate the level of advancement of an individual’s disease.

To test the reliability of the SuStaIn model, we examined the consistency of subtype assignments on follow-up MRI data. The results supported the effectiveness of the disease progression model in subtyping and staging, as 95.2% of the individuals showed stable subtype assignments over time. This includes individuals who were consistently assigned to the same subtype, and those who progressed from the normal-appearing group to corresponding atrophy subtypes as the brain atrophy initiated in either prefrontal/somatomotor or limbic-related regions. Overall, the model demonstrated a subtyping capability as high as 95.2%. Staging reliability refers to the proportion of follow-up visits where individuals either advanced to a higher SuStaIn stage or remained at the same stage as baseline assessment. This model exhibited a staging reliability of 89.8%, which could be attributed to the lower probabilities of stage assignment in unstable-stage cases, making them more prone to being retrogressed to an earlier stage. The reason for the “subtype unstable” or “stage unstable” assignments in longitudinal assessments could be attributed to various factors, including technical issues that may lead to inconsistencies in the measured imaging features used to classify subtypes or stages. Moreover, our finding revealed progressive worsening of brain atrophy over time, with longer follow-up periods associated with greater changes in SuStaIn stage, reflecting more advanced disease progression.

There are several limitations to consider in future work. One limitation is the inherent heterogeneity of the ALS–FTD spectrum. Our clinical assessments were routinely collected clinical measures (e.g., ALSFRS-R, UMN) that largely did not differ across observed subtypes, but more detailed clinical exam or finer-grained motor measures may better identify how our observed patterns may relate to clinical heterogeneity in future studies. Our study specifically focused on individuals associated with TDP-43 proteinopathies. This selective focus may restrict the generalizability of SuStaIn model in capturing the full extent of heterogeneity within the ALS–FTD spectrum, including bvFTD due to a tauopathy or atypical form of AD. Another limitation is the lack of sampling from important regions including spinal cord and brainstem, which play crucial roles in the pathophysiology of ALS. This limitation may partially explain why approximately 40% of ALS individuals were assigned to the normal-appearing group without apparent brain atrophy, as their pathology might be predominantly localized to the spinal cord and brainstem. The absence of data from these regions may mask important changes occurring specifically in spinal cord and brainstem, thereby restricting our ability to fully comprehend the underlying neurodegenerative processes in ALS. Moreover, while the SuStaIn modeling has generally only been applied to a single neuroimaging modality (e.g., positron emission tomography or MRI), we fully expect that future uses of multimodal imaging that incorporates diffusion MRI (e.g., reduced cerebrospinal tract integrity), spinal cord imaging, or muscle imaging may further improve the granularity of our observed subtypes. Incorporating additional data from spinal cord and brainstem could then potentially unveil empirical evidence of a spinal/brainstem-predominant subtype. Furthermore, while we demonstrated distinct subtypes within the ALS–FTD spectrum in this study using neuroimaging, the application of modern neuroimaging methods to clinical practice faces many challenges. Nonetheless, it is important to highlight that these findings provide a foundation for future studies aimed at uncovering the biological underpinnings of our reported subtypes. Future investigations should address these limitations to gain a more comprehensive understanding of the ALS–FTD spectrum. Moreover, it will be important for future studies to cross-validate our fitted SuStaIn model using another independent neuroimaging dataset but these validations are currently challenging given the lack of samples of phenotypically well-characterized, autopsy- or genetically confirmed TDP-43 proteinopathies. Also, in the absence of independent validation, our observed subtypes are hypothesis-generating to further evaluate additional mechanisms (e.g., RNA transcriptomics) that may drive heterogeneity across the ALS–FTD spectrum.

## Conclusions

In general, we utilized the SuStaIn model to gain a deeper understanding of the heterogeneity within the progressive processes of the ALS–FTD spectrum. We demonstrated two distinct spatiotemporal subtypes of cortical atrophy with varying clinical, genetic and neuropathological profiles, which shed light on the intricate progression patterns and heterogeneity of the ALS–FTD spectrum. This data-driven disease progression modelling method provides a valuable tool for individual classification and staging, paving the way for precision medicine in the field.

### Supplementary Information

**Additional file 1. Table S1**. Differences in volumes of brain regions classified to each cluster at baseline MRI in all individuals with ALS–FTD spectrum disorder relative to matched controls. **Table S2.** Comparison of baseline characteristics between subtypes in individuals with ALS/ALS–FTD. **Table S3.** Comparison of baseline characteristics between subtypes in individuals with bvFTD. **Table S4.** Longitudinal consistency of subtype assignments.** Fig. S1** Comparison of volumetric measurements between groups at baseline adjusting for SuStaIn stage. **Fig. S2** Correlations between SuStaIn stage and cognitive profiles. **Fig. S3** Longitudinal change of grey matter volume.

## Data Availability

All data used in this study are available upon reasonable request and approval from the Penn Neurodegenerative Data Sharing Committee. Requests may be submitted using a webform request: https://www.pennbindlab.com/data-sharing.

## Abbreviations

AD: Alzheimer’s disease
ALS: Amyotrophic lateral sclerosis
ALSFRS-R: Revised ALS Functional Rating Scale
BASC: Bootstrap Analysis of Stable Clusters
BNT: Boston Naming Test
bvFTD: Behavioral variant frontotemporal dementia
ECAS: Edinburgh Cognitive Assessment Scale
FTLD: Frontotemporal lobar degeneration
FTLD-TDP: FTLD due to TDP-43 proteinopathies
ALS-TDP: ALS with TDP-43 proteinopathies
GRN: Progranulin
MME: Metalloendopeptidase
MMSE: Mini-Mental Status Examination
PBAC: Philadelphia Brief Assessment of Cognition
PUMNS: Penn Upper Motor Neuron Score
SuStaIn: Subtype and Stage Inference
TBK1: TANK-binding kinase 1
TARDBP: TAR DNA-binding protein
