# Supplementary material for: Novel data-driven subtypes and stages of brain atrophy in the ALS–FTD spectrum
Source: Transl Neurodegener. 2023 Dec 7;12:57. doi: 10.1186/s40035-023-00389-3 (PMC10701950; doi:10.1186/s40035-023-00389-3)
Supplement: Supplementary file 1 — Additional file 1. Table S1. Differences in volumes of brain regions classified to each cluster at baseline MRI in all individuals with ALS–FTD spectrum disorder relative to matched controls. Table S2. Comparison of baseline characteristics between subtypes in individuals with ALS/ALS–FTD. Table S3. Comparison of baseline characteristics between subtypes in individuals with bvFTD. Table S4. Longitudinal consistency of subtype assignments. Fig. S1 Comparison of volumetric measurements between groups at baseline adjusting for SuStaIn stage. Fig. S2 Correlations between SuStaIn stage and cognitive profiles. Fig. S3 Longitudinal change of grey matter volume. [file 40035_2023_389_MOESM1_ESM.docx]

**Additional file 1**

### Table S1 Differences in volumes of brain regions classified to each cluster at baseline MRI in all individuals with ALS-FTD spectrum disorder relative to matched controls

| Region (Schaefer_Tian atlas labels) | t values | FDR-corrected p value | Cluster label |
| --- | --- | --- | --- |
| lh_SalVentAttnB_PFCl_1 | -9.01 | 9.11e-17 | 1 |
| lh_SalVentAttnB_PFCmp_1 | -8.92 | 1.72e-16 | 1 |
| lh_ContA_PFCl_1 | -7.76 | 3.31e-13 | 1 |
| lh_ContB_PFClv_1 | -7.91 | 1.28e-13 | 1 |
| lh_DefaultB_PFCd_1 | -7.40 | 3.51e-12 | 1 |
| lh_DefaultB_PFCv_2 | -9.02 | 9.11e-17 | 1 |
| rh_SalVentAttnB_PFCl_1 | -8.66 | 9.22e-16 | 1 |
| rh_SalVentAttnB_PFCmp_1 | -8.67 | 9.22e-16 | 1 |
| rh_ContA_PFCl_1 | -9.53 | 2.75e-18 | 1 |
| rh_ContA_PFCl_2 | -7.99 | 7.48e-14 | 1 |
| rh_ContB_PFCld_1 | -6.59 | 4.71e-10 | 1 |
| rh_DefaultA_PFCd_1 | -6.45 | 1.03e-09 | 1 |
| rh_DefaultB_PFCd_1 | -5.12 | 1.14e-06 | 1 |
| rh_DefaultB_PFCv_2 | -9.96 | 1.12e-19 | 1 |
| lh_SomMotA_1 | -1.27 | 0.24 | 2 |
| lh_SomMotA_2 | -2.19 | 0.04 | 2 |
| lh_SomMotB_Cent_1 | -5.82 | 3.40e-08 | 2 |
| rh_SomMotA_1 | -3.73 | 0.0004 | 2 |
| rh_SomMotA_2 | -2.97 | 0.005 | 2 |
| rh_SomMotA_4 | -2.11 | <0.05 | 2 |
| rh_SomMotB_Cent_1 | -8.28 | 1.21e-14 | 2 |
| rh_DorsAttnB_FEF_1 | -3.10 | 0.003 | 2 |
| lh_VisCent_ExStr_1 | 3.03 | 0.004 | 3 |
| lh_DorsAttnA_TempOcc_1 | -0.27 | 0.82 | 3 |
| lh_ContC_Cingp_1 | -0.19 | 0.87 | 3 |
| lh_DefaultC_PHC_1 | -1.93 | 0.07 | 3 |
| rh_VisCent_ExStr_1 | 2.14 | 0.04 | 3 |
| rh_DorsAttnA_TempOcc_1 | -2.11 | <0.05 | 3 |
| rh_ContC_Cingp_1 | -1.13 | 0.30 | 3 |
| rh_DefaultC_PHC_1 | -1.66 | 0.12 | 3 |
| rh_pTHA | -8.11 | 3.49e-14 | 4 |
| rh_aTHA | -8.25 | 1.38e-14 | 4 |
| rh_NAc | -6.98 | 4.47e-11 | 4 |
| rh_GP | -6.89 | 7.78e-11 | 4 |
| rh_PUT | -8.59 | 1.48e-15 | 4 |
| rh_CAU | -3.97 | 0.0002 | 4 |
| lh_pTHA | -7.30 | 6.36e-12 | 4 |
| lh_aTHA | -8.68 | 9.22e-16 | 4 |
| lh_NAc | -8.16 | 2.61e-14 | 4 |
| lh_GP | -6.03 | 1.08e-08 | 4 |
| lh_PUT | -8.40 | 5.17e-15 | 4 |
| lh_CAU | -4.31 | 3.99e-05 | 4 |
| lh_DorsAttnB_FEF_1 | -4.77 | 5.34e-06 | 5 |
| lh_SalVentAttnA_FrMed_1 | -4.68 | 8.02e-06 | 5 |
| lh_ContA_PFCl_2 | -5.76 | 4.52e-08 | 5 |
| lh_DefaultA_PFCd_1 | -4.80 | 4.85e-06 | 5 |
| lh_DefaultB_PFCl_1 | -5.80 | 3.66e-08 | 5 |
| rh_SalVentAttnA_FrMed_1 | -5.95 | 1.63e-08 | 5 |
| lh_DorsAttnB_PostC_1 | -1.48 | 0.16 | 6 |
| lh_DorsAttnB_PostC_2 | 0.36 | 0.78 | 6 |
| lh_DorsAttnB_PostC_3 | -0.69 | 0.54 | 6 |
| lh_SalVentAttnA_ParOper_1 | -2.17 | 0.04 | 6 |
| lh_SalVentAttnA_ParMed_1 | -4.59 | 1.16e-05 | 6 |
| lh_ContC_pCun_1 | -1.07 | 0.32 | 6 |
| rh_SomMotA_3 | -0.06 | 0.96 | 6 |
| rh_DorsAttnB_PostC_1 | -1.49 | 0.16 | 6 |
| rh_SalVentAttnA_ParMed_1 | -2.90 | 0.006 | 6 |
| rh_ContA_IPS_1 | 0.29 | 0.82 | 6 |
| lh_DefaultB_Temp_2 | -4.83 | 4.35e-06 | 7 |
| lh_DefaultB_IPL_1 | -5.05 | 1.54e-06 | 7 |
| lh_TempPar_1 | -3.93 | 0.0002 | 7 |
| rh_DorsAttnA_ParOcc_1 | -2.31 | 0.03 | 7 |
| rh_SalVentAttnB_IPL_1 | -4.72 | 6.80e-06 | 7 |
| rh_ContB_IPL_1 | -3.27 | 0.002 | 7 |
| rh_DefaultA_IPL_1 | -3.65 | 0.0005 | 7 |
| rh_TempPar_2 | -5.73 | 4.98e-08 | 7 |
| rh_TempPar_3 | -4.17 | 6.92e-05 | 7 |
| lh_VisCent_ExStr_2 | -2.57 | 0.02 | 8 |
| lh_VisCent_Striate_1 | 3.40 | 0.001 | 8 |
| lh_VisCent_ExStr_3 | -2.88 | 0.006 | 8 |
| lh_VisPeri_ExStrInf_1 | 1.59 | 0.14 | 8 |
| lh_VisPeri_StriCal_1 | 0.26 | 0.82 | 8 |
| lh_VisPeri_ExStrSup_1 | -0.17 | 0.88 | 8 |
| lh_DorsAttnA_ParOcc_1 | -2.07 | <0.05 | 8 |
| lh_DefaultC_Rsp_1 | -2.22 | 0.04 | 8 |
| rh_VisCent_ExStr_2 | -2.24 | 0.04 | 8 |
| rh_VisCent_ExStr_3 | -1.57 | 0.14 | 8 |
| rh_VisPeri_StriCal_1 | 3.41 | 0.001 | 8 |
| rh_VisPeri_ExStrInf_1 | -1.88 | 0.08 | 8 |
| rh_VisPeri_ExStrSup_1 | -1.16 | 0.29 | 8 |
| rh_DefaultC_Rsp_1 | -2.11 | <0.05 | 8 |
| rh_HIP | -6.37 | 1.56e-09 | 9 |
| rh_AMY | -7.32 | 5.54e-12 | 9 |
| lh_HIP | -7.93 | 1.09e-13 | 9 |
| lh_AMY | -7.61 | 8.61e-13 | 9 |
| lh_SomMotB_Aud_1 | -4.75 | 5.88e-06 | 10 |
| lh_SomMotB_S2_1 | -2.74 | 0.01 | 10 |
| lh_SomMotB_S2_2 | -3.28 | 0.002 | 10 |
| rh_SomMotB_Aud_1 | -4.24 | 5.30e-05 | 10 |
| rh_SomMotB_S2_1 | -3.73 | 0.0004 | 10 |
| rh_SomMotB_S2_2 | -5.28 | 5.06e-07 | 10 |
| rh_SalVentAttnA_ParOper_1 | -5.42 | 2.57e-07 | 10 |
| lh_SalVentAttnA_Ins_2 | -10.00 | 9.09e-20 | 11 |
| lh_LimbicB_OFC_1 | -10.91 | 1.46e-22 | 11 |
| lh_DefaultA_PFCm_1 | -9.51 | 2.88e-18 | 11 |
| lh_DefaultB_PFCv_1 | -9.12 | 4.87e-17 | 11 |
| rh_SalVentAttnA_Ins_1 | -10.73 | 3.65e-22 | 11 |
| rh_LimbicB_OFC_1 | -11.50 | 2.29e-24 | 11 |
| rh_ContB_PFClv_1 | -10.38 | 5.15e-21 | 11 |
| rh_DefaultA_PFCm_1 | -9.26 | 1.82e-17 | 11 |
| rh_DefaultB_PFCv_1 | -10.88 | 1.46e-22 | 11 |
| lh_SalVentAttnA_Ins_1 | -8.47 | 3.41e-15 | 12 |
| lh_LimbicA_TempPole_1 | -6.97 | 4.65e-11 | 12 |
| lh_LimbicA_TempPole_2 | -4.95 | 2.40e-06 | 12 |
| lh_DefaultB_Temp_1 | -5.95 | 1.63e-08 | 12 |
| rh_LimbicA_TempPole_1 | -6.46 | 9.70e-10 | 12 |
| rh_ContB_Temp_1 | -7.03 | 3.46e-11 | 12 |
| rh_TempPar_1 | -5.68 | 6.63e-08 | 12 |
| lh_DorsAttnA_SPL_1 | -1.77 | 0.10 | 13 |
| lh_ContA_IPS_1 | -0.28 | 0.82 | 13 |
| lh_ContC_pCun_2 | -0.28 | 0.82 | 13 |
| lh_DefaultA_pCunPCC_1 | 0.94 | 0.39 | 13 |
| rh_DorsAttnA_SPL_1 | -1.62 | 0.13 | 13 |
| rh_DorsAttnB_PostC_2 | -0.07 | 0.95 | 13 |
| rh_ContC_pCun_1 | -2.55 | 0.02 | 13 |
| rh_DefaultA_pCunPCC_1 | -0.66 | 0.55 | 13 |

### Table S2 Comparison of baseline characteristics between subtypes in individuals with ALS/ALS-FTD

|  | S1  *n* = 74 | S2  *n* = 33 | S0  *n* = 43 | Missing data | *P*_S1vsS2_ | *P*_S0vS1_ | *P*_S0vsS2_ |
| --- | --- | --- | --- | --- | --- | --- | --- |
| Age at MRI, years | 58.7 (10.9) | 62.0 (9.5) | 60.8 (9.3) | 0.0% | 0.13 | 0.27 | 0.61 |
| Sex, male% | 44 (59.5%) | 18 (54.6%) | 24 (55.8%) | 0.0% | 0.63 | 0.70 | 0.91 |
| Education, years | 17.2 (14.0) | 15.2 (2.7) | 15.2 (2.6) | 0.0% | 0.80 | 0.83 | 0.94 |
| Disease duration, months^a^ | 24.2 (199.5) | 26.2 (122.3) | 28.1 (126.9) | 0.0% | 0.96 | 0.76 | 0.86 |
| Diagnostic delay, months | 22.3 (21.5) | 33.3 (36.2) | 18.9 (21.0) | 0.0% | 0.24 | 0.26 | 0.05 |
| Clinical phenotypes | *n* = 74 | *n* = 33 | *n* = 43 | 0.0% | **0.03** | **0.0001** | **<0.0001** |
| ALS | 48 (64.9%) | 14 (42.4%) | 41 (95.4%) | - | - | - | - |
| ALS-FTD | 26 (35.1%) | 19 (57.6%) | 2 (4.7%) | - | - | - | - |
| Symptom onset site | *n* = 68 | *n* = 31 | *n* = 43 | 5.3% | **0.004** | 0.14 | **<0.0001** |
| LMN | 45 (66.2%) | 16 (51.6%) | 31 (72.1%) | - | - | - | - |
| UMN | 14 (1.5%) | 2 (6.5%) | 11 (25.6%) | - | - | - | - |
| Cognitive | 9 (13.2%) | 13 (41.9%) | 1 (2.3%) | - | - | - | - |
| PUMNS | 10.3 (7.6) | 8.5 (7.9) | 9.3 (8.2) | 14.0% | 0.30 | 0.47 | 0.63 |
| ALSFRS-R | 34.2 (6.6) | 34.8 (6.6) | 35.7 (7.6) | 6.0% | 0.54 | 0.17 | 0.48 |
| Progression index | 0.5 (0.4) | 0.6 (0.4) | 0.5 (0.4) | 6.0% | 0.52 | 0.59 | 0.33 |
| King’s stage | *n* = 67 | *n* = 31 | *n* = 43 | 6.0% | 0.46 | **0.02** | **0.005** |
| Stage 1 | 9 (13.4%) | 1 (3.2%) | 17 (39.5%) | - | - | - | - |
| Stage 2 | 24 (35.8%) | 11 (35.5%) | 9 (20.9%) | - | - | - | - |
| Stage 3 | 25 (37.3%) | 14 (45.2%) | 12 (27.9%) | - | - | - | - |
| Stage 4 | 9 (13.4%) | 5 (16.1%) | 5 (11.6%) | - | - | - | - |
| Median^a^ | 3.0 (3.0) | 3.0 (3.0) | 2.0 (3.0) | 6.0% | 0.24 | **0.04** | **0.009** |
| MMSE | 26.7 (3.9) | 23.8 (5.9) | 28.2 (2.2) | 7.3% | 0.07 | **0.01** | **0.001** |
| ECAS total | 97.2 (25.4) | 93.3 (23.5) | 113.9 (9,7) | 61.3% | 0.58 | **0.02** | **0.02** |
| ALS specific score | 71.8 (20.0) | 69.9 (17.5) | 85.5 (8.3) | - | 0.59 | **0.01** | **0.02** |
| ALS non-specific score | 25.5 (6.4) | 23.4 (7.7) | 28.4 (2.5) | - | 0.49 | 0.24 | 0.15 |
| PBAC total | 73.2 (12.1) | 66.7 (16.0) | 81.9 (3.8) | 54.7% | 0.20 | **0.0004** | **0.005** |
| Executive scale | 9.0 (4.1) | 9.3 (3.6) | 12.9 (2.4) | - | 0.97 | **0.0002** | **0.006** |
| Language scale | 17.2 (3.2) | 15.2 (4.1) | 18.6 (0.6) | - | **0.01** | **<0.05** | **<0.0001** |
| Visual scale | 16.5 (1.9) | 16.3 (2.6) | 17.7 (0.6) | - | 0.83 | **0.006** | 0.07 |
| Memory scale | 13.6 (4.2) | 10.5 (6.2) | 14.8 (3.3) | - | 0.11 | 0.24 | **0.03** |
| Behavioral scale | 17.0 (2.0) | 15.4 (2.7) | 18.0 (0.0) | - | **0.02** | **0.007** | **<0.0001** |
| BNT | 25.9 (4.7) | 16.8 (7.3) | 27.7 (1.9) | 48.0% | **<0.0001** | 0.21 | **<0.0001** |
| Animal fluency task | 15.0 (6.8) | 9.7 (7.1) | 20.0 (5.6) | 36.7% | **0.007** | **0.007** | **<0.0001** |
| Letter fluency task | 10.0 (5.4) | 7.5 (5.2) | 13.2 (4.4) | 6.7% | **0.03** | **0.001** | **<0.0001** |
| Digit forward span | 6.3 (1.3) | 6.1 (1.1) | 7.2 (1.1) | 15.3% | 0.47 | **0.002** | **0.0006** |
| Digit backward span | 4.4 (1.6) | 3.8 (1.7) | 4.8 (1.2) | 16.0% | 0.20 | 0.13 | **0.007** |
| SuStaIn stage | 6.5 (6.6) | 10.2 (7.7) | 0 (0.0) | 0.0% | **0.004** | - | - |

Data are presented as mean (standard deviation) for the continuous variables, and as number (frequency) for the categorical variables. King’s stage is also presented as median. Missing data indicates the percentage of individuals with missing data. ^a^ data presented as median (range).

*S0* Normal-appearing group, *S1* Prefrontal/Somatomotor-predominant subtype, *S2* Limbic-predominant subtype, *ALS* amyotrophic lateral sclerosis, *ALS-FTD* amyotrophic lateral sclerosis-frontotemporal degeneration, *LMN* lower motor neuron, *UMN* upper motor neuron, *PUMNS* Penn Upper Motor Neuron Score, *ALSFRS-R* revised ALS Functional Rating Scale, *MMSE* Mini-Mental Status Examination, *ECAS* Edinburgh Cognitive Assessment Scale, *PBAC* Philadelphia Brief Assessment of Cognition, *BNT* Boston naming test, *SuStaIn* Subtype and Stage Inference.

### Table S3 Comparison of baseline characteristics between subtypes in individuals with bvFTD

|  | S1  *n* = 42 | S2  *n* = 14 | Missing data | *P*_S1vsS2_ |
| --- | --- | --- | --- | --- |
| Age at MRI, years | 62.1 (6.7) | 65.5 (9.4) | 0.0% | 0.51 |
| Sex, male% | 25 (59.5%) | 10 (71.4%) | 0.0% | 0.43 |
| Education, years | 16.3 (2.9) | 15.8 (2.6) | 0.0% | 0.52 |
| Disease duration, months^a^ | 33.4 (118.1) | 70.9 (180.6) | 0.0% | **0.001** |
| Diagnostic delay, months | 41.0 (25.7) | 64.1 (47.1) | 0.0% | 0.05 |
| MMSE | 24.0 (5.0) | 25.4 (4.7) | 3.5% | 0.23 |
| PBAC Total | 60.4 (15.1) | 57.2 (22.6) | 33.3% | 0.70 |
| Executive scale | 6.9 (3.6) | 7.9 (3.5) | - | 0.44 |
| Language scale | 15.0 (3.1) | 12.8 (5.9) | - | 0.52 |
| Visual scale | 13.9 (4.7) | 15.3 (5.5) | - | 0.15 |
| Memory scale | 12.3 (5.2) | 10.3 (6.6) | - | 0.44 |
| Behavioral scale | 12.4 (4.2) | 10.8 (5.3) | - | 0.43 |
| BNT | 23.7 (4.5) | 18.0 (9.1) | 8.8% | **0.03** |
| Animal fluency task | 11.0 (5.6) | 11.5 (7.4) | 3.5% | 0.86 |
| Letter fluency task | 6.9 (4.7) | 9.1 (4.2) | 7.0% | 0.07 |
| Digit forward span | 5.8 (1.5) | 5.9 (1.4) | 10.5% | 0.85 |
| Digit backward span | 3.2 (1.4) | 4.1 (1.6) | 10.5% | 0.08 |
| Genetic pathogenic variants^b^ | *n* = 42 | *n* = 14 | 0.0% | **-** |
| *C9orf72* | 25 (59.5%) | 5 (35.7%) | - | 0.14 |
| *GRN* | 11 (26.2%) | 0 (0.0%) | - | **<0.05** |
| *TBK1* | 0 (0.0%) | 1 (7.1%) | - | 0.25 |
| *TARDBP* | 0 (0.0%) | 2 (14.3%) | - | 0.06 |
| FTLD/ALS-TDP^c^ | *n* = 18 | *n* = 9 | 52.6% | 0.05 |
| Type A | 11 (61.1%) | 3 (33.3%) | **-** | **-** |
| Type B/E | 6 (33.3%) | 3 (33.3%) | - | - |
| Type C | 0 (0.0%) | 3 (33.3%) | - | - |
| Non-specific | 1 (5.6%) | 0 (0.0%) | - | - |
| SuStaIn stage | 15.6 (7.2) | 16.8 (6.2) | 0.0% | 0.56 |

Data are presented as mean (standard deviation) for the continuous variables, and as number (frequency) for the categorical variables. Missing data indicates the percentage of individuals with missing data. ^a^ data presented as median (range); ^b^ number of individuals underwent genetic screening; ^c^ number of individuals underwent neuropathological examination.

*S0* Normal-appearing group, *S1* Prefrontal/Somatomotor-predominant subtype, *S2* Limbic-predominant subtype, *bvFTD* behavioral variant frontotemporal degeneration, *MMSE* Mini-Mental Status Examination, *ECAS* Edinburgh Cognitive Assessment Scale, *PBAC* Philadelphia Brief Assessment of Cognition, *BNT* Boston naming test, *FTLD/ALS-TDP* frontotemporal lobar degeneration or amyotrophic lateral sclerosis with TDP-43 inclusions, *SuStaIn* Subtype and Stage Inference.

### Table S4 Longitudinal consistency of subtype assignments

| Subtype at baseline | Subtype at follow-up visits | | |
| --- | --- | --- | --- |
|  | **Normal-appearing group** | **Prefrontal/Somatomotor-predominant subtype** | **Limbic-predominant subtype** |
| Normal-appearing group | 8 (66.7%) | 2 (16.7%) | 2 (16.7%) |
| Prefrontal/Somatomotor-predominant subtype | 0 (0.0%) | 35 (92.1%) | 3 (7.9%) |
| Limbic-predominant subtype | 0 (0.0%) | 0 (7.7%) | 12 (100.0%) |

Data are presented as number (frequencies of follow-up visits assigned to each group).


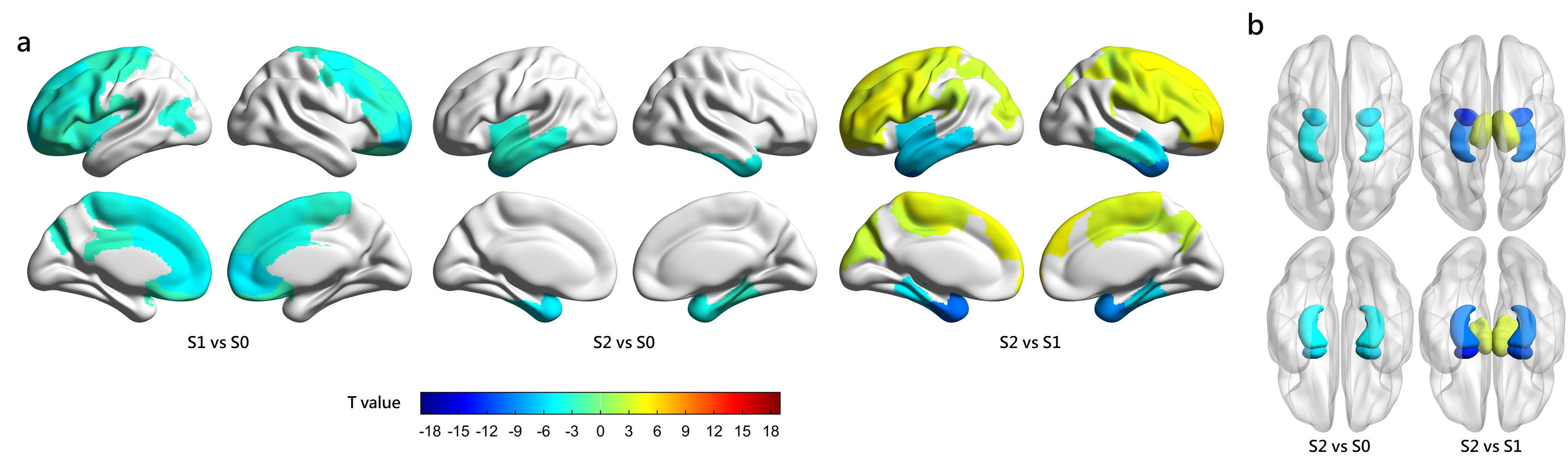


### Fig. S1 Comparison of volumetric measurements between groups at baseline adjusting for SuStaIn stage. (a) Cortical volumetric differences between groups at baseline. (b) Subcortical volumetric differences between groups at baseline. Only results with a threshold at FDR-corrected p value < 0.05 were shown. Cool colors indicate more cortical atrophy in the former group than the latter one, while warm colors indicate more cortical atrophy in the latter group than the former one. *S0* Normal-appearing group, *S1* Prefrontal/Somatomotor-predominant subtype, *S2* Limbic-predominant subtype


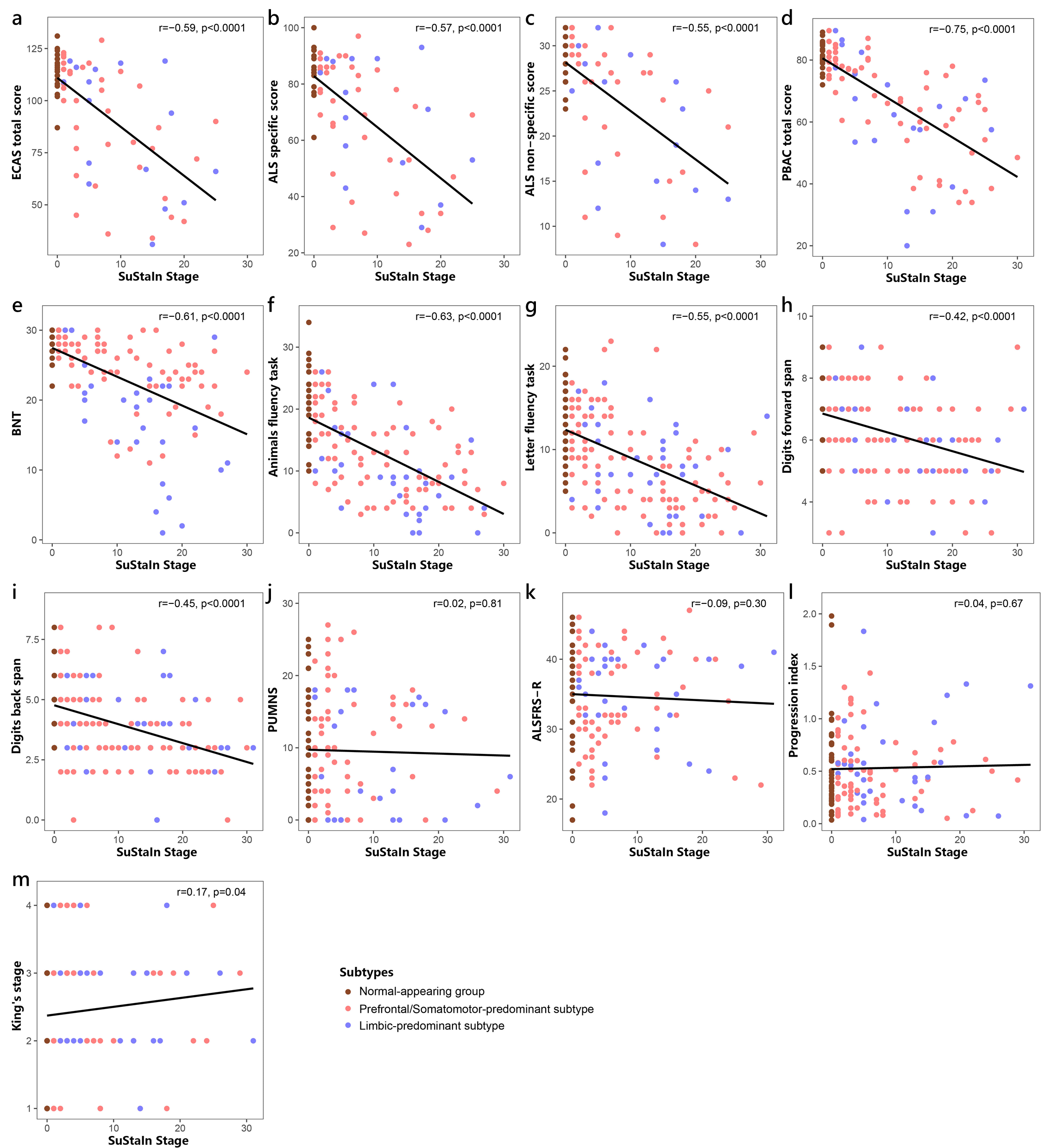


### Fig. S2 Correlations between SuStaIn stage and cognitive profiles. Correlations between SuStaIn stage and (a) ECAS total score, (b) ALS specific score, (c) ALS non-specific score, (d) PBAC total score, (e) BNT, (f) Animal fluency task, (g) Letter fluency task, (h) Digits forward span, (i) Digits back span, (j) PUMNS, (k) ALSFRS-R, (l) progression index, (m) King’s stage. *ECAS* Edinburgh Cognitive Assessment Scale, *PBAC* Philadelphia Brief Assessment of Cognition, *BNT* Boston naming test, *PUMNS* Penn Upper Motor Neuron Score, *ALSFRS-R* Revised ALS Functional Rating Scale, *SuStaIn* Subtype and Stage Inference


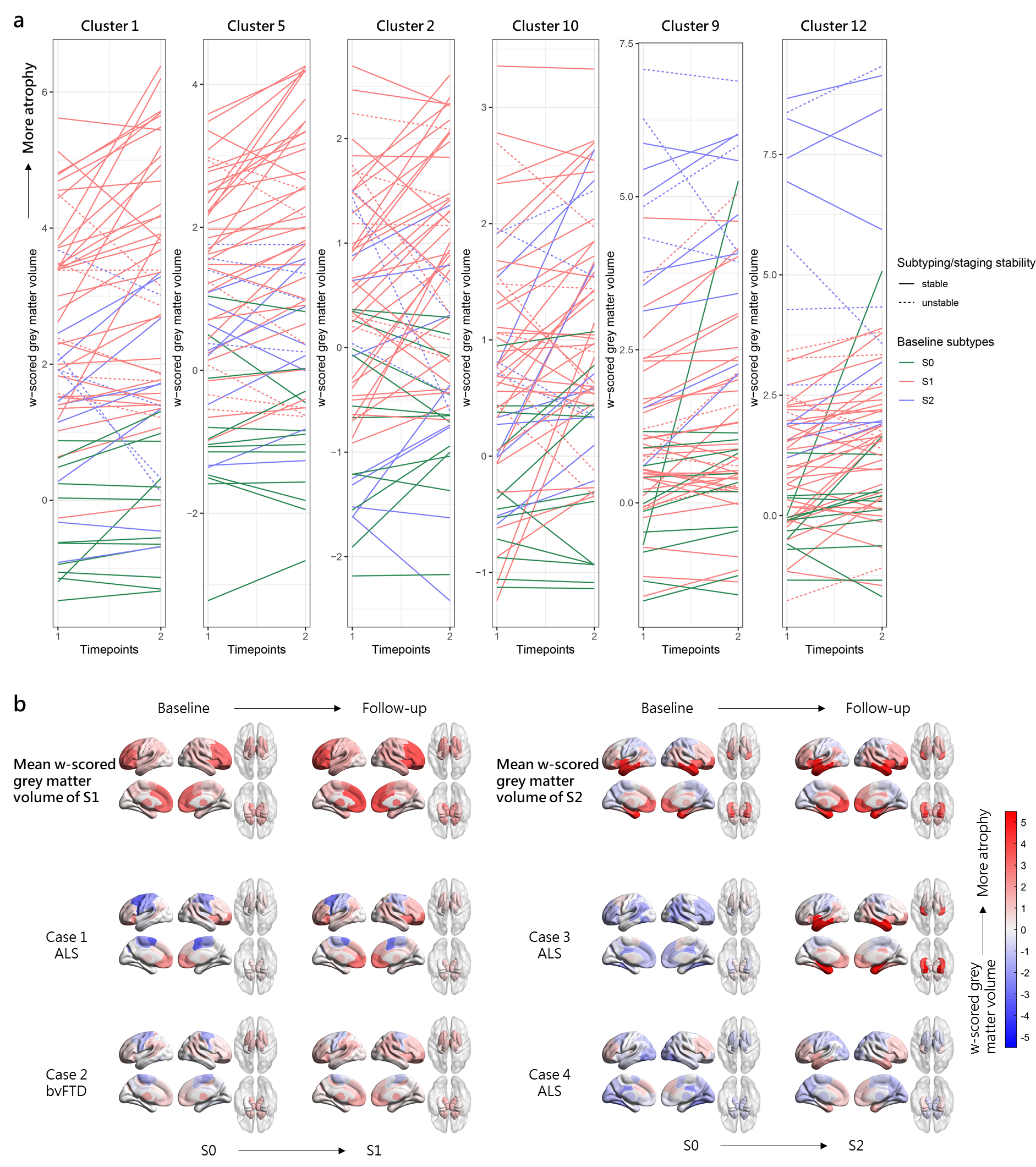


### Fig. S3 Longitudinal change of grey matter volume. (a) Longitudinal change of grey matter volume in BASC-identified clusters at individual level. Higher w-scored grey matter volume indicates more atrophy in our model. Timepoint 1 and 2 refers to the baseline MRI scan and follow-up MRI scan. The dashed line represents individuals whose follow-up visit is classified as either subtype unstable or stage unstable. (b) Brain heatmap showing the grey matter volume. The first row displays the mean w-scored grey matter volume of the two subtypes at baseline and follow-up visits. The second and third rows represent four cases within the normal-appearing group at baseline. In the follow-up, case 1 and case 2 progressed to subtype 1, while case 3 and case 4 progressed to subtype 2. *S0* Normal-appearing group, *S1* Prefrontal/Somatomotor-predominant subtype, *S2* Limbic-predominant subtype
